# Systematic analysis of tRNA transcription unit deletions in *E. coli* reveals insights into tRNA gene essentiality and cellular adaptation

**DOI:** 10.1038/s41598-024-73407-7

**Published:** 2024-10-15

**Authors:** Sanja Tiefenbacher, Valérie Pezo, Philippe Marlière, Tania M. Roberts, Sven Panke

**Affiliations:** 1 Bioprocess Laboratory, Department of Biosystems Science and Engineering, ETH Zurich, 4056 Basel, Switzerland; 2 Genoscope, Génomique Métabolique, Institut François Jacob, CEA, CNRS, Univ Evry, Université Paris-Saclay, 91057 Evry, France; 3 TESSSI, The European Syndicate of Synthetic Scientists and Industrialists, 75002 Paris, France

**Keywords:** tRNA gene deletion, *E. coli* gene, Deletion collection, tRNA pool, Genomics, Biotechnology, Genetics, Microbiology, Molecular biology

## Abstract

Transfer ribonucleic acids (tRNAs) are essential for protein synthesis, decoding mRNA sequences into amino acids. In *E. coli* K-12 MG1655, 86 tRNA genes are organized in 43 transcription units (TUs) and the essentiality of individual tRNA TUs in bacterial physiology remains unclear. To address this, we systematically generated 43 *E. coli* tRNA deletion strains in which each tRNA TU was replaced by a kanamycin resistance gene. We found that 33 TUs are not essential for survival, while 10 are essential and require the corresponding TU to be provided on plasmid. The analysis revealed *E. coli*’s tolerance to alterations in tRNA gene copy number and the loss of non-essential tRNAs, as most strains exhibited minimal to no growth differences under various conditions compared to the parental strain. However, deletions *metZWV*, *alaWX* and *valVW* led to significant growth defects under specific conditions. RNA-seq analysis of *∆alaWX* and *∆valVW* revealed upregulation of genes involved in translation and pilus assembly. Our results provide valuable insights into tRNA dynamics and the cellular response to tRNA TU deletions, paving the way for deeper understanding of tRNA pool complexity.

**Supplementary Information:**

The online version contains supplementary material available at 10.1038/s41598-024-73407-7.

## Introduction

Transfer ribonucleic acids (tRNAs) play a central role in protein synthesis by decoding the nucleotide sequence of mRNA into the primary amino acid sequence of a protein. In *E. coli* K-12 MG1655, 86 tRNA genes are organized in 43 transcription units (TUs), either as individual genes or combined with other mRNA, tRNA or rRNA genes^1^. The tRNA pool consists of various tRNA isoacceptor families, in which each family member carries a different anticodon sequence, but all charge the same amino acid via decoding by Watson Crick base pairing plus Wobble interactions^2^. In *E. coli* many tRNAs demonstrate redundancy, meaning that their tRNA anticodon can be encoded by more than one gene copy. While the sequence of tRNAs within the same isoacceptor family are identical or highly similar^3^, their promoter and terminator sequences differ when located in different TUs.

The abundance of tRNAs is strongly correlated with codon usage across multiple growth rates^4^. Additionally, during the elongation phase of translation, the availability of tRNAs significantly influences the efficiency and accuracy of gene translation^4–6^. Numerous studies have explored the functional roles of specific tRNAs^7–12^ and investigated how changes in tRNA concentration impact protein synthesis^13^, solubility^14,15^, tRNA charging patterns^16^, and transcriptome modulation^17^.

Despite the importance of tRNAs, it remains unclear whether single tRNA TUs can be deleted from the chromosome. The EcoCyc database^18,19^ and the Keio knockout collection^20^ lack data on the essentiality of tRNA TUs in *E. coli*. Additionally, the essentiality of tRNA TUs during the construction of a reduced *E. coli* genome remained unexplored^21^. As a result, there is a lack of comprehensive empirical data to investigate the impact of tRNA deletions, leaving uncertainty about whether they can be deleted at all and if so, what potential negative effects are associated with such deletions.

To fill this gap, a recent study in *E. coli* MG1655 examined the redundancy of individual tRNA genes in cellular physiology by deleting some of the redundant gene copies and studying their effect on cellular survival and translation under diverse growth conditions^22^. The findings showed that the redundancy in tRNA pools is beneficial in nutrient-rich environments but becomes costly under nutrient limitations, confirming similar observations from a comprehensive study previously conducted in yeast^23^.

By carefully considering the redundancy of tRNA genes and wobble rules for decoding the genetic code (Table S1, S2), it is possible to predict the essentiality of each tRNA TU (Table S3). To systematically validate these predictions, we generated 43 *E. coli* tRNA deletion strains in which each tRNA TU was replaced by a kanamycin resistance gene. We found that 33 TUs are not essential for survival, while 10 are essential and require their corresponding TU to be provided on plasmid. Although two TUs did not align with our initial predictions, our analysis revealed *E. coli’s* tolerance to both changes in tRNA gene copy number and the loss of non-essential tRNAs, as most strains exhibit minimal to no growth differences in varying growth conditions compared to the parental strain. However, some deletions led to aberrant growth phenotypes, including *∆alaWX* and *∆valVW*. We used RNA-seq to examine whether a removal of the *alaWX* and *valVW* tRNA TUs respectively led to a potential imbalance in tRNA levels triggering a common cellular response. We observed that the deletion of these tRNA TUs resulted in upregulation of genes involved in translation processes and pilus assembly. Notably, only a small subset of genes was regulated in a similar manner. While we could mostly predict the essentiality of tRNA TUs, the impact of each deletion and tRNA complementation on cell viability was not predictable. Therefore, our results provide valuable insights into these dynamics and serve as a resource for future investigations of tRNA pool and its involvement in cellular physiology.

## Results

### Generation of tRNA TU deletion strains and assessment of their essentiality

We systematically generated a set of tRNA deletions in *E. coli* MG1655 (DE3) by individually deleting each of the 43 tRNA TUs. In each strain, a single tRNA TU was replaced by a kanamycin resistant cassette (KanR) using Lambda Red homologous recombination^24^.

**Fig. 1:**
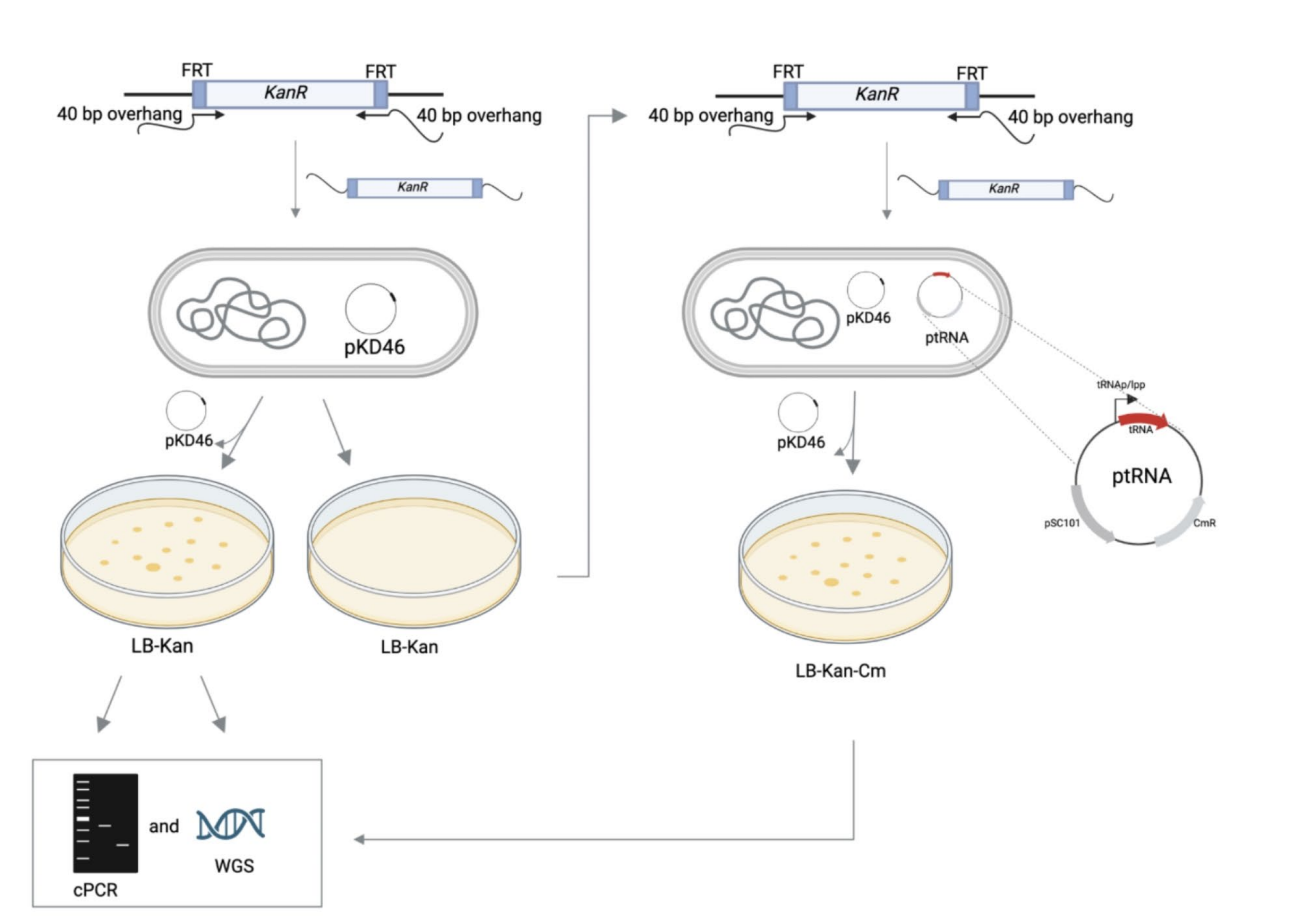
Generation of tRNA TU deletion strains and determination of their essentiality. (A) Kanamycin resistant gene (KanR) is PCR amplified with appropriate overhangs. In each strain, a single tRNA TU is replaced by the KanR using homologous recombination. ptRNA plasmid is a low-copy plasmid that complements a wild-type tRNA TU targeted for deletion under the control of either its natural or the *lpp* promoter. Upon deletion of a non-essential tRNA TU, the cell remains viable, and colonies are formed on LB-Kan agar plates. Upon deletion of an essential tRNA TU, the cell becomes inviable, and no colonies form on LB-Kan agar plates. The tRNA TU must be complemented by the plasmid (ptRNA) carrying the corresponding TU before removing the TU from the chromosome. Finally, tRNA knockout strains are confirmed by colony PCR (cPCR) and further validated by whole genome sequencing (WGS). LB-Lysogeny broth, Kan-kanamycin, Cm-chloramphenicol, pKD46-Lambda Red recombinase expression plasmid.

By considering the redundancy of tRNA genes and the wobble rules for decoding, we predicted the essentiality of each tRNA TU. To validate these predictions, we conducted experimental analysis. If a TU resisted disruption even after three attempts, it was identified as a candidate for an essential TU. On the other hand, if the TU could be disrupted within three attempts, it was classified as non-essential (Fig. 1; Table 1).

**Table 1:** Essential tRNA TUs predicted and identified in *E. Coli* MG1655 (DE3).

Experimentally identified tRNA transcription unit	Anticodon (5’ → 3’)	Predicted essential tRNA element (Anticodon 5’ → 3’)
*gltU-aspT-trpT* (*rrnC*)	UUC-GUC-ACC	*trpT* (ACC)
*argX-hisR-leuT-proM*	CCG-GUG-CAG-UGG	*hisR* (GUG), *proM* (UGG)
*thrU-tyrU-glyT-thrT*	UGU-GUA-UCC-GGU	*thrU* (UGU), *glyT* (UCC)
*argU*	UCU	*argU* (UCU)
*metT-leuW-glnUW-metU-glnVX*	CAU-UAG-UUG-UUG-CAU-CUG-CUG	*metTU* (CAU), *leuW* (UAG), *glnUW* (UUG), *glnVX* (CUG)
*serT*	UGA	*serT* (UGA)
*lysT-valT-lysW-valZ-lysYZQ*	UUU-UAC-UUU-UAC-UUU-UUU-UUU	- ^a^
*glyW-cysT-leuZ*	GCC-GCA-UAA	*cysT* (GCA), *leuZ* (UAA)
*argQZYV-serV*	ACG-ACG-ACG-ACG-GCU	*argQZYV* (ACG), *serV* (GCU)
*ilex*	CAU	- ^b^

^a^ 5/6 UUU anticodons are in this operon.

^b^ 1/2 CAU anticodons are present in this operon.

We found that 33 TUs could be deleted without the need for complementation on a plasmid, indicating that these TUs are non-essential. As expected, all non-essential tRNA TUs belong to multi-copy tRNA isoacceptor families. However, the remaining 10 TUs could only be deleted in the presence of a complementing tRNA plasmid expressing the TU of interest, suggesting that these 10 TUs are essential for *E. coli* survival. Complementing tRNA plasmid (for the individual plasmid names see Table S4) is a low-copy plasmid carrying the corresponding wild-type tRNA TU under the control of its natural promoter. As an exception, *argU* and *rrnC* TUs could only be successfully removed from the chromosome after their natural promoter had been exchanged by the strong *lpp* promoter on the complementing plasmid.

Among the essential tRNA TUs, we identified three units that carry the single tRNA gene for each of the following amino acids: tryptophan (*trpT* (ACG)), histidine (*hisR* (GUG)), and cysteine (*cysT* (ACG)). Additionally, we identified five units that belong to multicopy tRNA family containing all anticodons for a specific tRNA family (*proM* (UGG), *thrU* (UGU), *glyT* (UCC), *argU* (UCU), *leuW* (UAG), *serT* (UGA), *leuZ* (UAA), *serV* (GCU), *metTU* (CAU), *glnUW* (UUG), *glnVX* (CUG), *argQZYV* (ACG)). Additionally, *lysT-valT-lysW-valZ-lysYZQ* could only be deleted in the presence of a complementing plasmid, despite identical valine and lysine tRNAs present in *valUXY-lysV*, indicating that *valUXY-lysV* alone could not sustain the growth of *E. coli.* Surprisingly, the *ileX* TU also appears to be essential despite the presence of an *ileY* on the chromosome, which only differs from *ileX* at nucleotide positions 6 and 67, which do not play an important role in tRNA structure^25,26^. In all cases, successful deletion of the TU of interest was confirmed by the absence of respective tRNA TU from the locus by colony PCR (cPCR) (Figure S1) and further validated by whole-genome sequencing of the deletion strains.

### Characterization of cellular fitness of tRNA deletion strains

To examine the cellular fitness of the individual tRNA deletion strains, we compared growth in minimal and rich media in combination with different temperatures (Figure S2). The growth of each knockout strain was qualitatively compared to the growth of the parental strain under the same conditions. Based on these comparisons, the strains were classified as not impaired, slightly impaired, or very impaired.

**Fig. 2:**
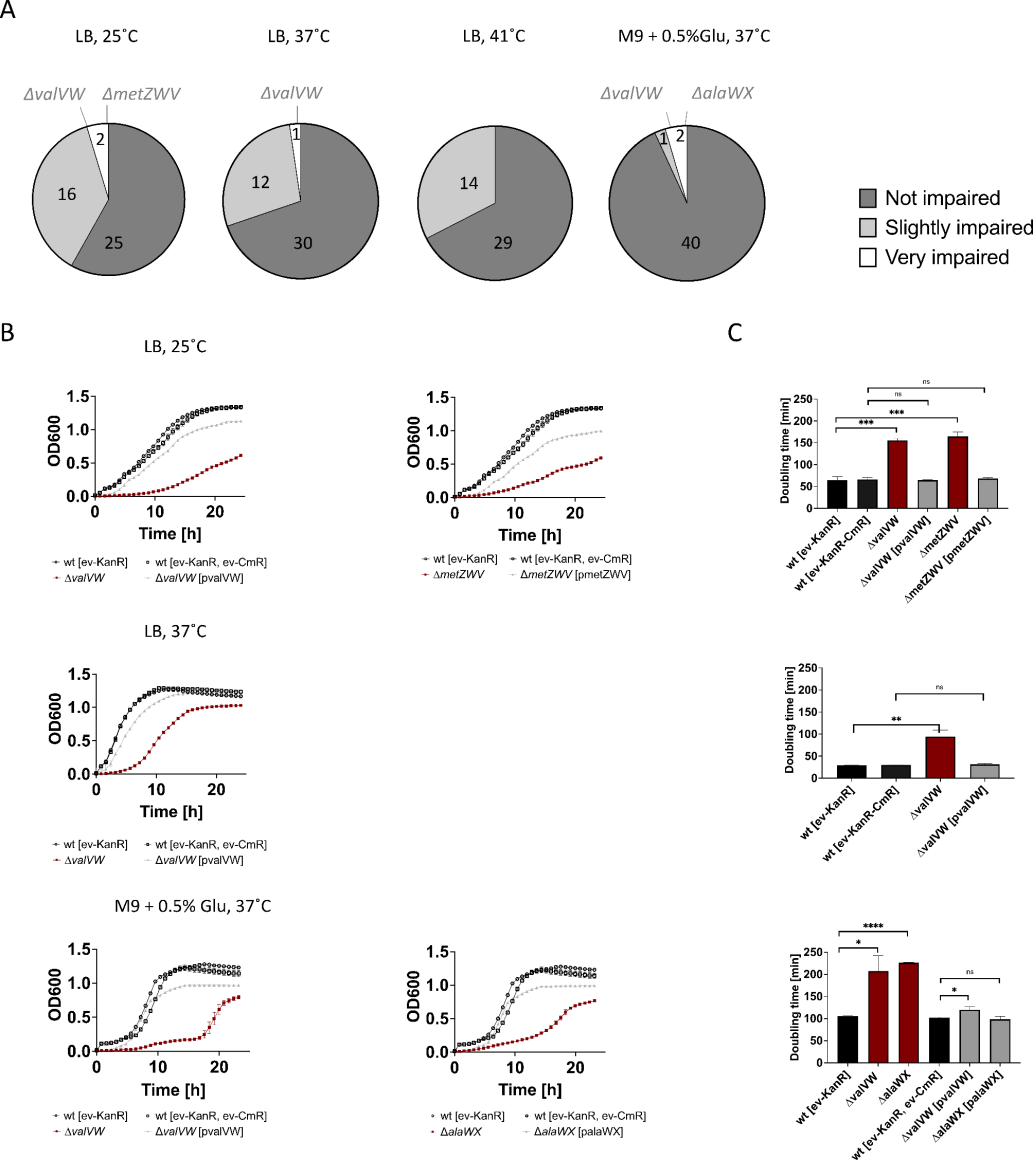
Examination of the growth of wild-type (wt) and tRNA deletion mutants under diverse growth conditions. Parental strain and tRNA deletion mutants were grown in the specified medium and temperature and growth was monitored by measuring OD_600_. (**A**) The number of strains with a given phenotype in different growth conditions is shown. (**B**) The growth curves of wt with empty pSEVA271 (wt-KanR; black circles), wt with empty pSEVA271 and pSEVA361 (wt-KanR-CmR; black squares), and very impaired non-essential knockout strains with (grey triangles) and without (red quares) the tRNA complementing plasmid. (**C**) Doubling times of corresponding strains and conditions were determined in the exponential growth phase. All growth experiments are performed in quadruplicates. An unpaired t-test was used to compare doubling times between tRNA knockout mutants and the wt (ns (non-significant), ** (p-value ≤ 0.01), *** (p-value ≤ 0.001), **** (p-value ≤ 0.0001)). Error bars indicate the standard deviation of the replicates. LB-Lysogeny broth, Kan-kanamycin, Cm-chloramphenicol, M9-minimal medium, Glc-glucose, ev-empty vector.

Most strains with an essential TU removed and sustained with a corresponding complementing tRNA plasmid showed minimal to no growth impairment under specific conditions. This suggests that the complementing tRNA plasmid was generally sufficient for optimal cell growth despite a difference in gene copy number. The majority (21/33) of non-essential tRNA deletion strains exhibited a similar growth phenotype to the parental strain, indicating the robustness of *E. coli* to tRNA TU deletion (Fig. 2A). However, the removal of *valVW*, *alaWX*, or *metZWV* resulted in a significant growth impairment under specific conditions. These strains were classified as very impaired. Despite retaining at least one copy of each tRNA gene family, these cells were unable to fully compensate for the loss of the tRNA TU by upregulating the remaining copies. However, growth was restored upon complementation with the corresponding tRNA TU on a plasmid (Fig. 2B, C), confirming that the observed growth defect was a result of tRNA loss. These results are consistent with prior studies, in which it was shown that growth impairment caused by tRNA gene copy removal can be rescued by introducing the corresponding wild-type tRNA on the plasmid^9,27,28^.

### Transcriptome profiling

To understand the cellular response to the disturbance of tRNA levels caused by the elimination of a tRNA TU, we conducted an extensive transcriptome analysis. We focused on the deletion of *alaWX* and *valVW* TUs as their removal resulted in impaired growth in a minimal medium at 37 °C. We hypothesized that there would be an overlap of the up or downregulation of specific genes as a result of disruptions of tRNA levels. In addition, we wanted to examine the overall cellular response from the individual deletions.

**Fig. 3:**
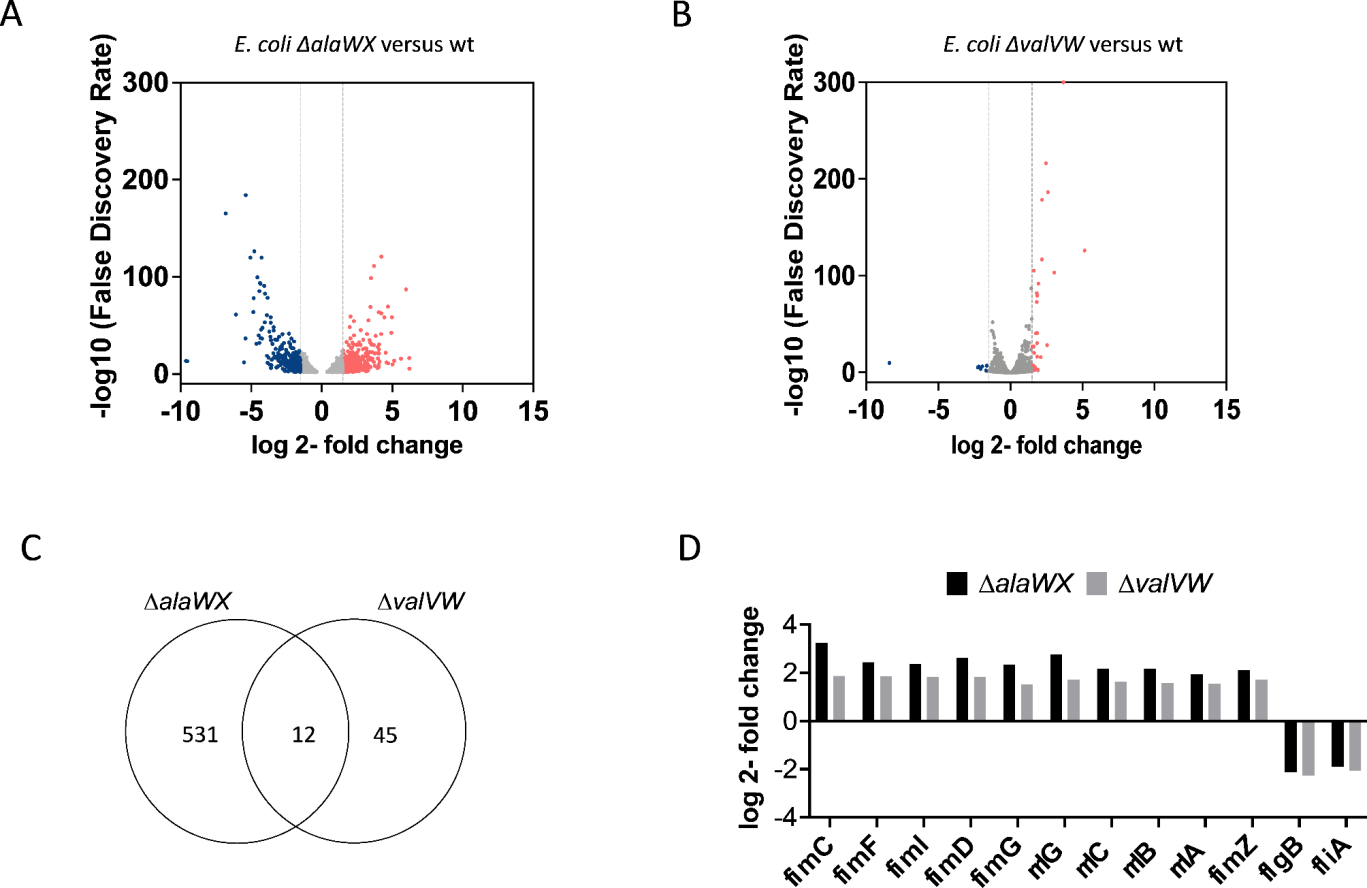
RNA-seq analysis reveals differentially expressed genes in response to perturbed tRNA levels. Wild-type (wt), *∆alaWX*, and *∆valVW* were grown in M9 + 0.5% glucose at 37 °C. Samples (*n* = 3) were harvested in the mid-log phase, total RNA was extracted, and RNA sequencing was performed. The differential expression of genes (DEGs) in *∆alaWX* (**A**) and *∆valVW* (**B**) versus wt was analyzed. Red and blue points mark differentially expressed genes respectively (genes that pass thresholds for false discovery rate < 0.05 and log2-fold change ≥ 1.5, ≤ -1.5). Grey points represent genes that did not meet the threshold and are thus categorized as non-differentially expressed genes. (**C**) The number of DEGs is shown for each strain, the overlap indicates the number of genes that were similarly regulated. These genes and their corresponding regulation are highlighted in (**D**).

We performed RNA-seq and differential gene expression (DGE) analysis to compare the transcriptomes of tRNA mutant strains with wild-type and considered genes that exhibited log2-fold change values ≥ + 1.5 or ≤-1.5 and false discovery rate (FDR) smaller than 0.05 as differentially expressed. Upon removal of the *alaWX* TU, we identified 530 differentially expressed genes, with 272 genes upregulated and 258 genes downregulated. Conversely, the removal of the *valVW* TU resulted in only 44 differentially expressed genes, with 37 genes upregulated, and 7 genes downregulated. We found 12 overlapping differentially expressed genes between the two samples (Fig. 3, Table S10-S13).

Within the 12 overlapping genes, 6 genes were involved in pilus assembly and protein folding. These genes included *fimC*,* fimF*,* fimI*,* fimD*,* fimG* (from the *fimAICDFGH* TU), and the gene for the transcriptional regulator *fimZ.* Four genes, specifically *rrlG*, *rrlC*, *rrlB*, and *rrlA* from the *rrnG*, *rrnC*, *rrnB*, and *rrnA* TUs respectively were upregulated. These genes encode 23S rRNAs, which play a crucial role in translation. In addition, *flgB* (from the *flgBCDEFGHIJ* TU) that forms the rod of the flagellar basal body, and *fliA* (from the *fliAZ* TU) encoding a minor sigma factor (σ^28^) responsible for the initiation of transcription of several genes involved in motility and flagellar synthesis were downregulated (Table 2). KEGG Orthology-Based Annotation System (KOBAS) pathway analysis of the differentially expressed genes in ∆*alaWX* and ∆*valVW* revealed the majority of perturbed pathways related to metabolism and biosynthesis. Notably, the “ribosome pathway” (KEGG pathway ko03010, which includes ribosomal proteins), was enriched in both *∆alaWX* and *∆valVW*. On the other hand, in *∆valVW* only the flagellar assembly pathway was depleted, whereas *∆alaWX* exhibited depletion of 12 different pathways (Table 3).

**Table 2:** List of commonly regulated DEGs in ∆*alaWX* and ∆*valVW* with corresponding log2- fold change (FC) and gene ontology biological process.

Gene ID	Gene description^a^	log2- FC		Gene ontology biological process
		*∆alaWX*	*∆valVW*	
*fimC*	type 1 fimbriae periplasmic chaperone	3.25	1.88	protein folding
*fimF*	type 1 fimbriae minor subunit FimF	2.44	1.86	pilus assembly
*fimI*	putative fimbrial protein FimI	2.37	1.84	pilus assembly
*fimD*	type I fimbriae usher protein	2.62	1.83	pilus assembly
*fimG*	type 1 fimbriae minor subunit FimG	2.34	1.52	pilus assembly
*fimZ*	putative LuxR family transcriptional regulator FimZ	2.12	1.71	transcription regulation (pilus assembly)
*rrlG*	23 S ribosomal RNA	2.76	1.71	translation
*rrlC*	23 S ribosomal RNA	2.16	1.63	translation
*rrlB*	23 S ribosomal RNA	2.17	1.58	translation
*rrlA*	23 S ribosomal RNA	1.93	1.54	translation
*flgB*	flagellar basal-body rod protein FlgB	-2.13	-2.26	bacterial-type flagellum-dependent cell motility
*fliA*	RNA polymerase sigma factor fliA	-1.89	-2.05	transcription initiation

^a^ Gene descriptions were retrieved from the NCBI.

**Table 3:** Revealing transcriptome alterations in *∆alaWX* and *∆valVW* through KEGG Orthology-based annotation system analysis (FDR < 0.05).

Genotype	Description	Observed gene count	Background gene count
*∆alaWX*	**Enriched pathways**		
	Histidine metabolism	7	8
	Biosynthesis of amino acids	18	117
	Ribosome	13	78
	Pyruvate metabolism	10	53
	Nitrotoluene degradation	4	7
	**Depleted pathways**		
	ABC transporters	27	172
	Citrate cycle (TCA cycle)	10	27
	Biosynthesis of siderophore group nonribosomal peptides	6	7
	Biosynthesis of antibiotics	26	210
	Oxidative phosphorylation	10	43
	Tryptophan metabolism	5	10
	Biotin metabolism	5	14
	Lysine degradation	5	17
	Arginine and proline metabolism	6	25
	Carbon metabolism	14	109
	Aminoacyl-tRNA biosynthesis	14	111
	Propanoate metabolism	7	39
*∆valVW*	**Enriched pathways**		
	Two-component system	8	166
	C5-branched dibasic acid metabolism	2	10
	Ribosome	4	78
	Valine, leucine, and isoleucine biosynthesis	2	16
	**Depleted pathways**		
	Flagellar assembly	4	37

The observed gene count is the number of genes from the input gene set linked to a particular pathway in the KEGG database. The background gene count is the number of genes in the reference set of genes linked to the same pathway.

The log2-fold changes of neither the remaining alanine nor the valine tRNA genes met the defined threshold of ≥ + 1.5 or ≤-1.5, thus excluding them from being classified as differentially expressed. Nevertheless, we analyzed these genes carefully, given their potential importance as integral components of the alanine and valine tRNA pools.

**Fig. 4:**
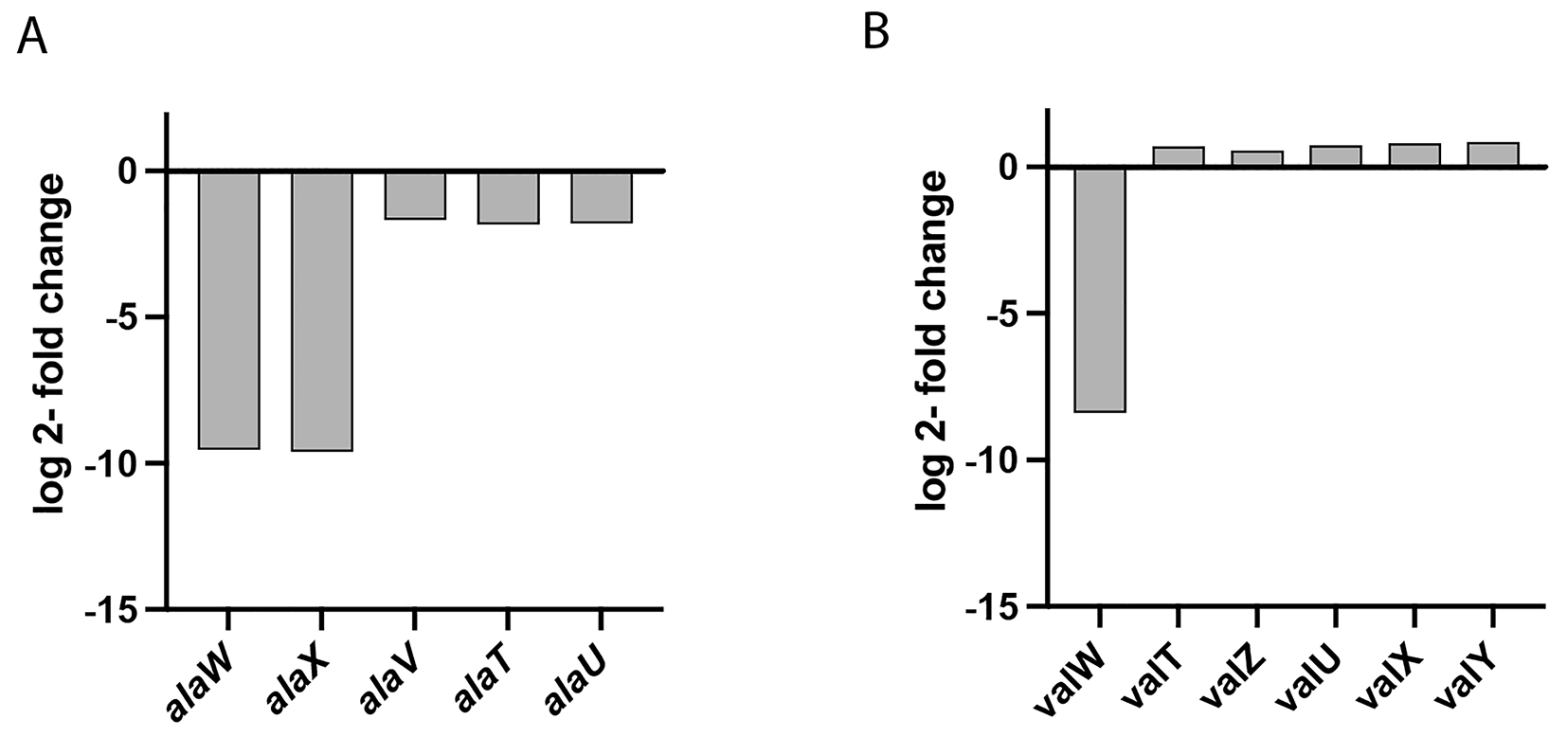
Effects of *alaWX* and *valVW* TU deletion on alanine and valine tRNA pools. Wild-type (wt), *∆alaWX* and *∆valVW* were grown in M9 + 0.5% glucose at 37 °C. Samples (*n* = 3) were harvested in the mid-log phase, total RNA was extracted, and RNA sequencing was performed. Expression levels of the remaining alanine (**A**) and valine (**B**) tRNAs in *∆alaWX* and *∆valVW* respectively are shown (FDR < 0.0001).

Based on the predicted matching pattern between codons and tRNAs, it is apparent that the cognate codon of tRNA *alaWX* (GGC), which is GCC, can also be translated using the *alaV*, *alaT*, and *alaU* (UGC) tRNAs through Wobble pairing (Figure S3). However, we did not observe any upregulation of the remaining alanine tRNA genes in *∆alaWX*; instead, they exhibited slight downregulation (Fig. 4A). On the other hand, the cognate codon of *valVW* (GAC), which is GUC, can also be translated using the remaining valine tRNAs (*valU*, *valX*, *valY*, *valT*, *valZ* (UAC)) through wobbling. Interestingly, the remaining valine genes in *∆valVW* were slightly upregulated compared to the wild-type strain (Fig. 4B). However, the expression of these backup genes was not sufficient to fully compensate for the loss of the removed TU in terms of growth in a minimal medium (Fig. 2).

## Discussion

We generated a comprehensive collection of *E. coli* tRNA deletion strains, where in each strain the individual tRNA TU is replaced with a kanamycin resistance gene flanked by FLP recognition target (FRT) sites. This design allows for potential excision of the FRT-flanked resistance gene using a plasmid expressing Flp recombinase^29^. Essential tRNA TUs were deleted in the presence of a plasmid complementing a corresponding missing TU with a pSC101 origin of replication and chloramphenicol resistance gene, making it compatible to be used together with pACYC and colE1-type plasmids. Whole genome sequencing confirmed that no gene duplications had occurred^30^.

This deletion collection revealed that tRNA TUs are dispensable under various growth conditions, suggesting that compensation within tRNA families varies depending on the condition. This indicates that the tRNA pool is dynamic and can adjust to meet cellular needs^23^. Complementation with a corresponding tRNA plasmid restored growth in impaired strains, suggesting that the observed growth defects were a result of tRNA level perturbations and that copy number affects growth, as reported previously^9,28^. Most tRNA TUs were successfully complemented on the plasmid with their natural promoters, but tRNAs from the *rrnC* TU and *argU* required strong *lpp* promoter for successful complementation. Attempts to complement these genes on a plasmid with a higher copy number but under the control of the natural promoter were unsuccessful (data not shown), suggesting that both *rrnC* and *argU* natural promoters may be under tight regulation and are not sufficient to drive appropriate expression of tRNA genes on the plasmid.

Studies in *E. coli* and yeast^22,23^, highlight that single base changes, the genomic context of each gene, or differences in promoter sequences play a role in functionality and expression. Similarly, we observed that the removal of *ileX* (CAU) required plasmid complementation, despite the presence of *ileY* (CAU), which differs from *ileX* by only 2 nucleotides. In contrast, the removal of *ileY* did not require plasmid complementation. Furthermore, the *lysT-valT-lysW-valZ-lysYZQ* TU was essential, despite identical tRNAs found in the *valUXY-lysV* TU, indicating that a single *lysV* copy was not sufficient for the cell survival, highlighting the importance of having multiple copies of some tRNA genes for proper cellular function.

Given the critical role of tRNA concentration in the translation rate of a single codon^14,32^ and mistranslation rates^33^, and the similar behavior of *∆alaWX* and *∆valVW* when grown in a minimal medium, we predicted that there may be a common pattern in the cellular response to tRNA level disruption. However, transcriptomic analysis revealed only 12 genes were commonly dysregulated. These genes were upregulated in translation and pilus assembly and downregulated in flagellar assembly. Although we did not study this in detail, previous studies have shown that the downregulation of genes involved in flagellar assembly can disrupt swimming motility due to translation inefficiencies^34,35^.

In contrast to the compensation observed among the rRNA TUs, where the loss of some rRNA TUs is compensated by the upregulation of backup gene copies^36^, we did not observe a similar compensatory effect in the remaining tRNA genes after the loss of *alaWX* and *valVW*.

The *alaWX* genes encode both GGC anticodon that decodes the frequently occurring GCC and GCU codons in *E. coli*. Similarly, the *valVW* encode the GAC anticodon that decodes the frequent GUC and GUU codons (Figure S3). Removal of *alaWX* and *valVW* restricts the decoding of all four alanine and valine codons to the modified uridine at position 34 in the remaining anticodons: UGC for alanine (cmo^5^U and mcmo^5^U) and UAC for valine (cmo^5^U)^37^. These mutants have also been previously observed with reduced viability, indicating that the remaining anticodons can read all four alanine or valine codons in a codon box, but not efficiently enough to minimize the growth impairment^38^.

In summary, we systematically created a complete set of single tRNA TU deletions to determine their dispensability and impact on cell viability. This deletion collection represents a valuable addition to the existing Keio collection^20^ and provides a resource to the scientific community to explore the complexities of translation and other essential cellular processes.

## Methods

### Chemicals and reagents

If not stated otherwise all chemicals and reagents were obtained from Sigma Aldrich (Buchs, Switzerland). Non-phosphorylated oligonucleotides were purchased from IDT (Coralville, IA, USA) and enzymes from New England Biolabs (Ipswich, MA, USA) or Invitrogen (Waltham, MA, USA). DNA extraction kits were obtained from Qiagen (Hilden, Germany) or Zymo Research (Irvine, CA, USA). PCRs were carried out using high-fidelity Phusion polymerase and colony PCRs (cPCRs) using Taq-polymerase. Sanger sequencing was performed at Microsynth (Balgach, Switzerland).

### Bacterial strains, plasmids, and oligonucleotides

All bacterial strains, plasmids, and DNA oligonucleotides used in this study are listed in Supplementary Tables 4–8. All tRNA knockout mutants were derived from the parental strain *E. coli* MG1655 (DE3)^39^.

### Growth media

*E. coli* MG1655 (DE3) and its derivatives were grown in LB-liquid or on LB-agar plates containing 1.5% bacto agar. Unless stated otherwise, M9 mineral medium^40^ (pH 7.4 adjusted with NaOH) was supplemented with 5 g L^− 1^ glucose and 100 µl L^− 1^ trace elements solution^41^. When required media were supplemented with antibiotics at the following concentrations: ampicillin (100 µg mL^− 1^), kanamycin (50 µg mL^− 1^), and chloramphenicol (25 µg mL^− 1^).

### Construction of tRNA knockout strains

All tRNA knockout strains were constructed by replacing tRNA TU with kanamycin resistant cassette flanked by FLP recognition target (FRT) by λ Red based recombineering^24^. Plasmid pKD4 was amplified using pairs of oligonucleotides listed in Table S6 resulting in a fragment containing the KanR cassette with 40 bp overhangs homologous to the genomic regions up- and downstream of the tRNA TU to be deleted. The PCR product was purified according to the manufacturer’s instructions. Next, *E. coli* MG1655 (DE3) harboring λ Red expression plasmid pKD46 were made electrocompetent. For this, cells were grown overnight in 2 mL of LB supplemented with ampicillin at 30˚C at 300 rounds per minute (rpm) with a shaking amplitude of 25 mm in a Kuhner shaker (Birsfelden, Switzerland). The culture was diluted 1:100 into 10 mL of fresh LB medium with ampicillin, grown at 30˚C and 200 rpm to an optical density at 600 nm (OD_600_) of 0.6, induced with 1mM L-arabinose (30 °C, 200 rpm), and then cells were harvested after 2 h by centrifugation (4,000 rcf, 4 °C, 10 min). Cells were washed three times with 10 mL of ice-cold dH_2_O, re-centrifuged in between (4,000 rcf, 4 °C, 10 min), and finally, the cell pellet was resuspended in ice-cold dH_2_O. An aliquot of 50 µl of cell suspension was used for electroporation. About 500 ng of the purified PCR fragment was added to the cells and the cells were subjected to electroporation with a micropulser (Bio-Rad, Hercules, CA, USA) at 1.8 kV in a cuvette with 1 mm gap (Cell Projects, Harrietsham, UK). After the electroporation, 950 µl of LB medium was added to the cells, followed by incubation at 37˚C and 200 rpm for 2 h. An aliquot of 100 µl of the cell suspension was plated on LB agar plates supplemented with kanamycin in order to select kanamycin resistant mutants. The remaining cells were spun down and plated on LB agar plates with kanamycin as well. Colonies that grew on LB/kanamycin were then tested for removal of the tRNA TU by colony PCR using the oligonucleotides listed in Table S7, and strains containing the desired deletion were stored as cryostocks with 20% glycerol (v/v) at -80 °C. When needed, the same protocol was performed with *E. coli* MG1655 (DE3) harboring pKD46 and a tRNA complementing plasmid expressing the tRNA TU that was to be deleted (ptRNA); chloramphenicol was added in required steps to retain the tRNA complementing plasmid. To verify the absence of the pKD46 plasmid, all strains were restreaked on LB-agar plates containing ampicillin and incubated overnight at 30˚C. The inability of strains to grow on ampicillin confirmed the loss of the plasmid.

### Construction of tRNA complementing plasmids

DNA sequences containing *argU* and *rrnC* tRNA genes under the control of *lpp* promoter were obtained by DNA synthesis (TWIST Bioscience, San Francisco, CA, USA) (Table S8). The remaining fragments required were amplified directly from the chromosome. All fragments for the tRNA complementing plasmid assembly were amplified using oligonucleotides listed in Table S9. Template plasmid (pSC101) was digested using DpnI for 2 h at 37˚C and PCR products were purified using a PCR purification kit. Next, the PCR fragments and template plasmid were ligated using Gibson assembly^42^ and the resulting mix was used to transform chemically competent *E. coli* DH5α cells. Individual clones were sequence verified by Sanger sequencing and then used to transform *E. coli* MG1655 (DE3) harboring pKD46.

### Whole-genome sequencing

Genomic DNA of *E. coli* was purified using Invitrogen genomic DNA preparation kit according to the manufacturer’s instructions (Waltham, MA, USA. The library preparation and sequencing were performed by Novogene (Cambridge, UK) using Illumina NovaSeq 6000 platform (insert size 250 bp, read length 150 bp). The obtained reads were assembled using Unicycler^43^. Genome assemblies were visualized using Bandage^44^. The assembled graphs were a direct output of Unicycler and Bandage and were not subjected to any further manual refinement. To confirm the absence of a tRNA TU from its genomic locus we searched for the presence of the kanamycin resistance gene in a corresponding genomic locus and the absence of the deleted tRNA TU sequence in the *de novo* assembled genome.

### Bacterial growth analysis

Biological clones were grown overnight at 37° in LB or M9 medium supplemented with either kanamycin or kanamycin and chloramphenicol. Overnight cultures were diluted 1:100 into 200 µl of fresh LB or M9 medium in a 96-well plate. Growth experiments were performed in an Infinite M200 plate reader (Tecan, Männedorf, Switzerland) at 25˚C, 37˚C, and 41˚C, respectively, under agitation (432 rpm, 1 mm amplitude), and bacterial growth was monitored by measuring the OD_600_. In total 4 biological replicates were grown and measured for each strain.

### Extraction of total RNA and RNA-sequencing

All RNA-seq experiments were performed in biological triplicates. Overnight cultures of* ∆alaWX*,* ∆valVW*, and the respective parental strain were diluted 1:100 into 50 mL of fresh M9 medium + 0.5% glucose and incubated at 37˚C and 200 rpm until they reached an OD_600_ of approximately 0.6. An aliquot of 3 mL of culture was immediately added to 6 mL of RNA-protect Bacteria Reagent (Qiagen, Hilden, Germany), briefly vortexed, and incubated for 5 min at room temperature. Bacterial cells were harvested by centrifugation (4,000 rcf, 10 min). The cell pellets were stored at -80˚C. The total RNA extraction and RNA-sequencing were performed by Genewiz (Germany) according to their standard protocol. Data including the normalized gene hit counts, log2-fold change values with corresponding p and padj values were obtained from Genewiz and afterward analyzed and visualized in GraphPad. All log2-fold change values in this manuscript are relative to the parental strain. Genes exhibiting a log2-fold change ≥ + 1.5 or ≤-1.5 together with a false discovery rate (FDR) below 0.05, were considered as differentially expressed. These differentially expressed genes were grouped into various gene ontology groups according to the EcoCyc^19^ database and were used for pathway enrichment analysis in KEGG Orthology-Based Annotation System (KOBAS) 3.0 ^45^. To determine the significance of pathway enrichment analysis a cut-off criterion of FDR < 0.05 was applied.

## Electronic supplementary material

Below is the link to the electronic supplementary material.

Supplementary Material 1

Supplementary Material 2

Supplementary Material 3

## Data Availability

The raw Illumina reads from RNA-Seq have been deposited in the NCBI SRA under the accession number PRJNA1126438. The raw reads from WGS sequencing can be found under the accession number PRJNA1128606.
