# Supplementary material for: Systematic analysis of tRNA transcription unit deletions in E. coli reveals insights into tRNA gene essentiality and cellular adaptation
Source: Sci Rep. 2024 Oct 15;14:24102. doi: 10.1038/s41598-024-73407-7 (PMC11480407; doi:10.1038/s41598-024-73407-7)
Supplement: Supplementary file 1 — Supplementary Material 1 [file 41598_2024_73407_MOESM1_ESM.docx]

**Supporting Information**

**Systematic Analysis of tRNA Transcription Unit Deletions in *E. coli* Reveals Insights into tRNA Gene Essentiality and Cellular Adaptation**

Sanja Tiefenbacher^1^, Valérie Pezo^2^, Philippe Marlière^3^, Tania M. Roberts^1^, Sven Panke^1*^

^1^ *Bioprocess Laboratory, Department of Biosystems Science and Engineering, ETH Zurich, 4056 Basel, Switzerland*

^2^ *Génomique Métabolique, Genoscope, Institut François Jacob, CEA, CNRS, Univ Evry, Université Paris-Saclay, 91057 Evry, France*

^3^ *TESSSI, The European Syndicate of Synthetic Scientists and Industrialists, 75002 Paris, France*

*corresponding author: sven.panke@bsse.ethz.ch

**Figure S 1**: Confirmation of chromosomic alterations of the E. coli MG1655 (DE3) strain. (A) Principle of the confirmation of removal of a tRNA transcription unit by PCR. Deletion of the indicated tRNA transcription unit was verified by colony PCR using oligonucleotides that bind to the flanking regions of a respective tRNA transcription unit before and after its replacement by a kanamycin resistance gene. (B) The expected size of the PCR products for the wild-type (wt) and knockout strains (KO) are highlighted with full and dotted arrows, respectively. C - control (no DNA template). The uncropped agarose gel images are provided in the Appendix.

Continuation on the next page.

**Figure S 2**: Examination of the growth of tRNA knockout strains (∆tRNA) under diverse growth conditions. The growth of wild-type (wt) and ∆tRNA strains was monitored by measuring OD_600_ while they were grown in the specified media and temperature. The growth curves of wt (black, circles) and tRNA knockout strains (red, squares) are shown. The growth of non-essential tRNA knockout strains is compared to wt with an empty vector pSEVA271 (KanR). The growth of essential knockouts is compared to the wt with pSEVA271 and pSEVA361 (CmR). Strains lacking essential transcription units and carrying a plasmid complementing the missing transcription unit are written in bold and underlined. Strains with slightly impaired growth are indicated by grey circles, and very impaired strains by red circles. Kanamycin was added to the media for non-essential knockout strains and wt [pSEVA271]. Chloramphenicol and kanamycin were added to the media for wt [pSEVA271, pSEVA361] and knockout strains with complementing tRNA plasmids. The complementing tRNA plasmids contain the corresponding wt tRNA transcription unit, regulated by its endogenous promoter or the lpp promoter. All growth experiments are performed in quadruplicates, and error bars represent the standard deviation of the replicates. LB-Lysogeny broth, M9-minimal medium, Glc-glucose.

**Figure S 3:** Translational system of alanine and valine in E. coli MG1655. (A) Genomic location of all alanine and valine tRNA genes in E. coli MG1655. (B) The predicted translational relationship between alanine and valine tRNAs and corresponding codons. Connections signify a theoretical match (solid black lines = Watson Crick pairing, dotted line = Wobble pairing). Anticodons and codons are written in 5’→ 3’ direction. ^1^ Codon usage frequency is derived from the reference 1. ^2^ Data is derived from the reference 2.

| 5’ base in anticodon | 3’ base in the codon |
| --- | --- |
| G | U / C |
| C | G |
| U | A / G / U / C |
| I | A / U / C |

**Table S 1:** Wobble base pairing for decoding of genetic code.

The table is derived from the reference 3.

**Table S 2:** Overview of tRNA genes, anticodons, and codon recognition pattern in E. coli MG1655 (codons recognized through wobble base pairing are shown in brackets).

| **Amino acid** | **tRNA gene** | **Anticodon [3’ → 5’]** | **Codon recognition [5’ → 3’]** | **Transcription unit** |
| --- | --- | --- | --- | --- |
| Alanine (Ala) | *alaX*  *alaW* | CGG  CGG | GCU  GCC | *alaX-alaW*  *alaX-alaW* |
|  | *alaV*  *alaT*  *alaU* | CGU  CGU  CGU | GCA, GCG, (GCU), (GCC) | *rrnH*  *rrnA*  *rrnD* |
| Arginine (Arg) | *argV*  *argQ*  *argZ*  *argY* | GCA  GCA  GCA  GCA | CGU, CGC, CGA | *argQ-argZ-argY-argV-serV*  *argQ-argZ-argY-argV-serV*  *argQ-argZ-argY-argV-serV*  *argQ-argZ-argY-argV-serV* |
|  | *argX* | GCC | CGG | *argX* |
|  | *argW* | UCC | AGG | *argW* |
|  | *argU* | UCU | AGA, (AGG) | *argU* |
| Asparagine (Asn) | *asnT*  *asnV*  *asnW*  *asnU* | UUG  UUG  UUG  UUG | AAC, (AAU) | *asnT*  *asnV*  *asnW*  *asnU* |
| Aspartic acid (Asp) | *aspT*  *aspU*  *aspV* | CUG  CUG  CUG | GAC, (GAU) | *rrnC*  *rrnH*  *aspV* |
| Cysteine (Cys) | *cysT* | ACG | UGC, (UGU) | *glyW-cysT-leuZ* |
| Glutamine (Gln) | *glnX*  *glnV* | GUC  GUC | CAG | *glnX-glnV-metU-glnW-glnU-leuW-metT glnX-glnV-metU-glnW-glnU-leuW-metT* |
|  | *glnW*  *glnU* | GUU  GUU | CAA, (CAG) | *glnX-glnV-metU-glnW-glnU-leuW-metT glnX-glnV-metU-glnW-glnU-leuW-metT* |
| Glutamic acid (Glu) | *gltW*  *gltU*  *gltT*  *gltV* | CUU  CUU  CUU  CUU | CAA, (CAG) | *rrnG*  *rrnC*  *rrnB*  *rrnE* |
| Glycine (Gly) | *glyU* | CCC | GGG | *glyU* |
|  | *glyV*  *glyX*  *glyY*  *glyW* | CCG  CCG  CCG  CCG | GGG GGC, (GGU) | *glyV-glyX-glyY*  *glyV-glyX-glyY*  *glyV-glyX-glyY*  *glyW-cysT-leuZ* |
|  | *glyT* | CCU | GGA, (GGG) | *thrU-tyrU-glyT-thrT* |
| Histidine (His) | *hisR* | GUG | CAC, (CAU) | *argX-hisR-leuT-proM* |
| Isoleucine (Ile) | *ileT*  *ileU*  *ileV* | UAG  UAG  UAG | AUC, (AUU) | *rrnA*  *rrnD*  *rrnH* |
|  | *ileX*  *ileY* | UAC  UAC | AUA | *ileX*  *ileY* |
| Leucine (Leu) | *leuX* | AAC | UUG | *leuX* |
|  | *leuT*  *leuV*  *leuP*  *leuQ* | GAC  GAC  GAC  GAC | CUG | *argX-hisR-leuT-proM*  *leuV-leuP-leuQ*  *leuV-leuP-leuQ*  *leuV-leuP-leuQ* |
|  | *leuU* | GAG | CUC | *leuU* |
|  | *leuZ* | AAU | UUA, (UUG) | *glyW-cysT-leuZ* |
|  | *leuW* | GAU | ACG, (CUU), (CUC), (CUG) | *glnX-glnV-metU-glnW-glnU-leuW-metT* |
| Lysine (Lys) | *lysV*  *lysT*  *lysW*  *lysY*  *lySZ*  *lysQ* | UUU  UUU  UUU  UUU  UUU  UUU | AAA, (AAG) | *valU-valX-valY-lysV*  *lysT-valT-lysW-valZ-lysY-lysZ-lysQ*  *lysT-valT-lysW-valZ-lysY-lysZ-lysQ*  *lysT-valT-lysW-valZ-lysY-lysZ-lysQ*  *lysT-valT-lysW-valZ-lysY-lysZ-lysQ*  *lysT-valT-lysW-valZ-lysY-lysZ-lysQ* |
| Methionine (Met) | *metZ ^a)^*  *metW ^a)^*  *metV ^a)^*  *metY ^a)^*  *metU*  *metT* | UAC  UAC  UAC  UAC  UAC  UAC | AUG | *metZ-metW-metV*  *metZ-metW-metV*  *metZ-metW-metV*  *metY*  *glnX-glnV-metU-glnW-glnU-leuW-metT*  *glnX-glnV-metU-glnW-glnU-leuW-metT* |
| Phenylalanine (Phe) | *pheU*  *pheV* | AAG  AAG | UUC, (UUU) | *pheU*  *pheV* |
| Proline (Pro) | *proK* | GGC | CCG | *proK* |
|  | *proL* | GGG | CCC, (CCU) | *proL* |
|  | *proM* | GGU | CCA, (CCU), (CCC), (CCG) | *argX-hisR-leuT-proM* |
| Serine (Ser) | *serU* | AGC | UCG | *serU* |
|  | *serV* | UCG | AGC, (AGU) | *argQ-argZ-argY-argV-serV* |
|  | *serX*  *serW* | AGG  AGG | UCC, (UCU) | *serX*  *serW* |
|  | *serT* | AGU | UCA, (UCU), (UCC), (UCG) | *serT* |
| Threonine (Thr) | *thrV*  *thrW* | UGC  UGC | ACG | *rrnD*  *thrW* |
|  | *thrT* | UGG | ACC, (ACU) | *thrU-tyrU-glyT-thrT* |
|  | *thrU* | UGU | ACA, (ACU), (ACC), (ACG) | *thrU-tyrU-glyT-thrT* |
| Tryptophane (Trp) | *trpT* | ACC | UGG | *rrnC* |
| Tyrosine (Tyr) | *tyrU*  *tyrV*  *tyrT* | AUG  AUG  AUG | UAC, (UAU) | *thrU-tyrU-glyT-thrT*  *tpr-tyrV-tyrT*  *tpr-tyrV-tyrT* |
| Valine (Val) | *valV*  *valW* | CAG  CAG | GUU, GUC, (GUA), (GUG) | *valV-valW*  *valV-valW* |
|  | *valU*  *valX*  *valY*  *valT*  *valZ* | CAU  CAU  CAU  CAU  CAU | GUA, GUG, (GUU), (GUC) | *valU-valX-valY-lysV*  *valU-valX-valY-lysV*  *valU-valX-valY-lysV*  *lysT-valT-lysW-valZ-lysY-lysZ-lysQ*  *lysT-valT-lysW-valZ-lysY-lysZ-lysQ* |
| Selenocysteine (Sec) | *selC* | UCA | UGA | *selC* |

^a)^ initiator tRNA

**Table S 3:** tRNA transcription units in E. coli and their predicted and experimentally determined essentiality. The transcription units are listed in the order they appear on the chromosome in a clockwise direction, starting from oriC.

| **tRNA transcription unit**  **[Anticodon 5’ → 3’]** | **Comment [Codon 5’ → 3’]** | **Predicted essentiality** | **Experimentally determined essentiality** |
| --- | --- | --- | --- |
| *gltU-trpT-aspT (rrnC)* (UUC-CCA-GUC) | UUC is present in 4 transcription units (*rrnG, rrnC, rrnB, rrnE*); *trpT* is the sole tryptophan tRNA; GUC is present in 3 transcription units (*rrnC, rrnH*, *aspV*) | Essential | Essential |
|  |  |  |  |
| *argX-hisR-leuT-proM*  (CCG-GUG-CAG-UGG) | CCG is the sole anticodon that reads CGG codon; *hisR* is the sole histidine tRNA, CAG is present in 2 transcription units; UGG is the sole anticodon that reads CCA | Essential | Essential |
|  |  |  |  |
| *ileT-alaT (rrnA)*  (GAU-UGC) | GAU and UCG are present in 3 transcription units, respectively (*rrnA, rrnD, rrnH*) | Non-essential | Non-essential |
|  |  |  |  |
| *gltT (rrnB)* (UUC) | UUC is present in 4 transcription units on the genome (*rrnG, rrnC, rrnB, rrnE*) | Non-essential | Non-essential |
|  |  |  |  |
| *thrU-tyrU-glyT-thrT* (UGU-GUA-UCC-GGU) | UGU is the sole anticodon that reads ACA codon; GUA is present in 2 transcription units (*thrU-tyrU-glyT-thrT, tyrT-tyrV*); UCC is the sole anticodon that reads GGA codon; GGU (*thrT*) and UGU (*thrU*) can read ACC codon. | Essential | Essential |
|  |  |  |  |
| *gltV (rrnE)* (UUC) | UUC is present in 4 transcription units (*rrnG, rrnC, rrnB, rrnE*) | Non-essential | Non-essential |
|  |  |  |  |
| *pheU* | GAA is present in two transcription units (*pheV, pheU*) | Non-essential | Non-essential |
| (GAA) |  |  |  |
| *glyV-glyY-glyX* (GCC-GCC-GCC) | GCC is present in two transcription units (*glyV-glyY-glyX, glyW-cysT-leuZ*) | Non-essential | Non-essential |
|  |  |  |  |
| *leuX* | CAA and UAA (*leuZ*) can read UUG codon | Non-essential | Non-essential |
| (CAA) |  |  |  |
| *leuQ-leuP-leuV* (CAG-CAG-CAG) | CAG is present in 2 transcription units *(leuQ-leuP-leuV, glyW-cysT-leuZ)* | Non-essential | Non-essential |
|  |  |  |  |
| *ileV-alaV-aspU (rrnH)* (GAU-UGC-GUC) | GAU and UGC are present in 3 transcription units respectively (*rrnA, rrnD, rrnH*); GUC is present in 3 transcription units (*rrnC, rrnH, aspV*) | Non-essential | Non-essential |
|  |  |  |  |
| *aspV* | GUC is present in 3 transcription units (*rrnC, rrnH, aspV*) | Non-essential | Non-essential |
| (GUC) |  |  |  |
| *thrW* | CGU is present in 2 transcription units (*thrW, rrnD*) | Non-essential | Non-essential |
| (CGU) |  |  |  |
| *argU* | UCU is the sole anticodon that reads AGA codon | Essential | Essential |
| (UCU) |  |  |  |
| *metT-leuW-glnUW-metU-glnVX* (CAU-UAG-UUG-UUG-CAU-CUG-CUG) | All 2 CAU anticodons are in this transcription unit; UAG is the sole anticodon that reads CUA codon; All glutamine anticodons UUG and CUG are in this transcription unit. | Essential | Essential |
|  |  |  |  |
| *lysT-valT-lysW-valZ-lysYZQ* (UUU-UAC-UUU-UAC-UUU-UUU-UUU) | *5/6 UUC anticodons are in this transcription unit. UUC (*lysV*) is not able to sustain *E. coli* growth and could only be removed from the chromosome after complementation on the plasmid. UAC is present in 2 transcription units (*lysT-valT-lysW-valZ-lysY-lysQ, valU-valX-valY-lysV*) | Non-essential | Essential |
|  |  |  |  |
| *serW* | GGA is present in 2 transcription units (*serW, serX*) | Non-essential | Non-essential |
| (GGA) |  |  |  |
| *serT* | UGA is the sole anticodon that reads UCA codon | Essential | Essential |
| (UGA) |  |  |  |
| *serX* | GGA is present in 2 transcription units (*serW, serX*) | Non-essential | Non-essential |
| (GGA) |  |  |  |
| *tyrTV* | GUA is present in 2 transcription units (*thrU-tyrU-glyT-thrT, tyrT-tyrV*) | Non-essential | Non-essential |
| (GUA-GUA) |  |  |  |
| *valVW* (GAC-GAC) | GAC and UAC anticodons can read all valine codons (GUU, GUC, GUA, GUG) | Non-essential | Non-essential |
|  |  |  |  |
| *glyW-cysT-leuZ* (GCC-GCA-UAA) | GCC is present in two transcription units (*glyV-glyY-glyX, glyW-cysT-leuZ*), GCA is the sole cysteine anticodon, UAA is the sole anticodon that reads UUA codon. | Essential | Essential |
|  |  |  |  |
| *serU* | CGA and CGU (*serT*) can read UCG codon | Non-essential | Non-essential |
| (CGA) |  |  |  |
| *asnT* | GUU comes in 4 transcription units (*asnT, asnV, asnW, asnU*) | Non-essential | Non-essential |
| (GUU) |  |  |  |
| *asnW* | GUU comes in 4 transcription units (*asnT, asnV, asnW, asnU*) | Non-essential | Non-essential |
| (GUU) |  |  |  |
| *asnU* | GUU comes in 4 transcription units (*asnT, asnV, asnW, asnU*) | Non-essential | Non-essential |
| (GUU) |  |  |  |
| *asnV* | GUU comes in 4 transcription units (*asnT, asnV, asnW, asnU*) | Non-essential | Non-essential |
| (GUU) |  |  |  |
| *proL* | GGG and UGG (*proM*) can read CCU and CCC codons | Non-essential | Non-essential |
| (GGG) |  |  |  |
| *argW* | CCU and UCU (*argU*) can read AGG codon | Non-essential | Non-essential |
| (CCU) |  |  |  |
| *alaWX* | GGC and UGC (*alaV, alaT, alaU*) can read GCU and GCC codons | Non-essential | Non-essential |
| (GGC-GGC) |  |  |  |
| *valUXY-lysV* (UAC-UAC-UAC-UUU) | UAC is present in 2 transcription units (*lysT-valT-lysW-valZ-lysY-lysQ, valU-valX-valY-lysV*) | Non-essential | Non-essential |
|  |  |  |  |
| *gltW (rrnG)* | UUC is present in 4 transcription units (*rrnG, rrnC, rrnB, rrnE*) | Non-essential | Non-essential |
| (UUC) |  |  |  |
| *ileY* | CAU is present in two transcription units (*ileX, ileY*) | Non-essential | Non-essential |
| (CAU) |  |  |  |
| *argQZYV-serV* (ACG-ACG-ACG-ACG-GCU) | All ACG anticodons are in this transcription unit. GCU is the sole anticodon that reads AGC and AGU | Essential | Essential |
|  |  |  |  |
| *metZWV* (CAU-CAU-CAU) | CAU anticodon is present in two transcription units (*metZ-metW-metV, metY*) | Non-essential | Non-essential |
|  |  |  |  |
| *glyU* | CCC and CCU (*thrU-tyrY-glyT-thrT*) can read GGG codon | Non-essential | Non-essential |
| (CCC) |  |  |  |
| *pheV* | GAA is present in two transcription units (*pheV, pheU*) | Non-essential | Non-essential |
| (GAA) |  |  |  |
| *ileX* (CAU) | CAU is present in two transcription units (*ileX, ileY*). * ileX could be removed from the genome, only after being complemented on the plasmid | Non-essential | Essential |
|  |  |  |  |
| *metY* (CAU) | CAU anticodon is present in two transcription units (*metZ-metW-metV*, *metY*) | Non-essential | Non-essential |
|  |  |  |  |
| *leuU* (GAG) | GAG and UAG (*metT-leuW-glnU-glnW-metU-glnV-glnX*) can read CUC codon | Non-essential | Non-essential |
|  |  |  |  |
| *ileU-alaU-thrV (rrnD)* (GAU-UGC-CGU) | GAU and UGC are present in 3 transcription units, respectively (*rrnA, rrnD, rrnH*); CGU and UGU (*thrU-tyrU-glyT-thrT*) can read ACG codon | Non-essential | Non-essential |
|  |  |  |  |
| *proK* | CGG and UGG (*argX-hisR-leuT-proM*) can read CCG codon | Non-essential | Non-essential |
| (CGG) |  |  |  |
| *selC* |  | Non-essential | Non-essential |
| (UCA) |  |  |  |

**Table S 4:** Plasmids used in this study.

| **Plasmid** | **Description** | **Source** |
| --- | --- | --- |
|  |  |  |
| pKD46 | Lambda red system under the control of ParaB promoter, R 101 temperature-sensitive origin of replication, ampicillin resistance gene | ^4^ |
| pKD4 | FRT-kanamycin-FRT cassette, R6K origin of replication | ^4^ |
| pSEVA271 | Empty backbone | ^5^ |
| pSEVA361 | Empty backbone | ^5^ |
| pSC101 | Expression vector, pSC101 origin of replication, chloramphenicol resistance gene | Lab collection |
| pST40 | pSC101 containing *lysT-valT-lysW-valZ-lysYZQ* under control of its natural promoter | This study |
| pST41 | pSC101 containing *metT-leuW-glnUW-metU-glnVX* under control of its natural promotor | This study |
| pST42 | pSC101 containing *serV-argVYZQ* under control of its natural promoter | This study |
| pST43 | pSC101 containing argX-hisR-leuT-proM under control of its natural promoter | This study |
| pST44 | pSC101 containing *thrU-tyrU-glyT-thrT* under control of its natural promoter | This study |
| pST45 | pSC101 containing *glyW-cysT-leuZ* under control of its natural promoter | This study |
| pST46 | pSC101 containing *ileX* under control of its natural promoter | This study |
| pST51 | pSC101 containing *serT* under control of its natural promoter | This study |
| pST52 | pSC101 containing *argU* unit under control of its natural promoter | This study |
| pST57 | pSC101 containing tRNA genes from *rrnC* under control of Ptac promoter | This study |
| pST58 | pSC101 containing tRNA genes from *rrnC* under control of its natural promoter | This study |
| pST72 | pSC101 containing *argU* under control of *lpp* promoter | This study |
| pST73 | pSC101 containing tRNA genes from *rrnC* under control of *lpp* promoter | This study |

**Table S 5:** Strains used in this study. KanR–kanamycin resistanc.

| **Name** | **Genotype** | **Source** |
| --- | --- | --- |
|  |  |  |
| *E. coli* DH5α | *fhuA2 lac(del)U169 phoA glnV44 Φ80' lacZ(del)M15 gyrA96 recA1 relA1 endA1 thi-1 hsdR17* | Invitrogen |
| *E. coli* MG1655 (DE3) | F-λ-rph-1 (λDE3) | ^6^ |
| ∆valUXY-lysV | *E. coli* MG1655 (DE3), *∆valUXY-lysV*::kan FRT+ | This study |
| ∆leuX | *E. coli* MG1655 (DE3), *∆leuX*::kan FRT+ | This study |
| ∆aspV | *E. coli* MG1655 (DE3), *∆aspV*::kan FRT+ | This study |
| ∆metZWV | *E. coli* MG1655 (DE3), *∆metZWV*::kan FRT+ | This study |
| ∆argW | *E. coli* MG1655 (DE3), *∆argW*::kan FRT+ | This study |
| ∆leuQPV | *E. coli* MG1655 (DE3), *∆leuQPV*::kan FRT*+* | This study |
| ∆thrW | *E. coli* MG1655 (DE3), *∆thrW*::kan FRT+ | This study |
| ∆proL | *E. coli* MG1655 (DE3), *∆proL*::kan FRT+ | This study |
| ∆tyrTV | *E. coli* MG1655 (DE3), *∆tyrTV*::kan FRT+ | This study |
| ∆glyU | *E. coli* MG1655 (DE3), *∆glyU*::kan FRT+ | This study |
| ∆serU | *E. coli* MG1655 (DE3), *∆serU*::kan FRT+ | This study |
| ∆leuU | *E. coli* MG1655 (DE3), *∆leuU*::kan FRT+ | This study |
| ∆asnV | *E. coli* MG1655 (DE3), *∆asnV*::kan FRT+ | This study |
| ∆proK | *E. coli* MG1655 (DE3), *∆proK*::kan FRT+ | This study |
| ∆asnW | *E. coli* MG1655 (DE3), *∆asnW*::kan FRT+ | This study |
| ∆serX | *E. coli* MG1655 (DE3), *∆serX*::kan FRT+ | This study |
| ∆lysTWYZQ-valTZ | *E. coli* MG1655 (DE3), *∆lysT-valT-lysW-valZ-lysYZQ*::kan FRT+ [ptRNA40] | This study |
| ∆serV-argVYZQ | *E. coli* MG1655 (DE3), *∆serV-argVYZQ*::kan FRT+ [ptRNA42] | This study |
| ∆argX-hisR-leuT-proM | *E. coli* MG1655 (DE3), *∆argX-hisR-leuT-proM*::kan FRT+ [ptRNA43] | This study |
| ∆thrU-tyrU-glyT-thrT | *E. coli* MG1655 (DE3), *∆thrU-tyrU-glyT-thrT*::kan FRT+ [ptRNA44] | This study |
| ∆glyW-cysT-leuZ | *E. coli* MG1655 (DE3), *∆glyW-cysT-leuZ*::kan FRT+ [ptRNA45] | This study |
| ∆alaWX | *E. coli* MG1655 (DE3), *∆alaWX*::kan FRT+ | This study |
| ∆glyVYX | *E. coli* MG1655 (DE3), *∆glyVYX*::kan FRT+ | This study |
| ∆asnU | *E. coli* MG1655 (DE3), *∆asnU*::kan FRT+ | This study |
| ∆ileY | *E. coli* MG1655 (DE3), *∆ileY*::kan FRT+ | This study |
| ∆asnT | *E. coli* MG1655 (DE3), *∆asnT*::kan FRT+ | This study |
| ∆selC | *E. coli* MG1655 (DE3), *∆selC*::kan FRT+ | This study |
| ∆metTU-leuW-glnUWVX | *E. coli* MG1655 (DE3), *∆metT-leuW-glnUW-metU-glnVX*::kan FRT+ [ptRNA41] | This study |
| ∆valVW | *E. coli* MG1655 (DE3), *∆valVW*::kan FRT+ | This study |
| ∆rrnA | *E. coli* MG1655 (DE3), *∆rrnA*::kan FRT+ | This study |
| ∆rrnB | *E. coli* MG1655 (DE3), *∆rrnB*::kan FRT+ | This study |
| ∆pheV | *E. coli* MG1655 (DE3), *∆pheV*::kan FRT+ | This study |
| ∆ileX | *E. coli* MG1655 (DE3), *∆ileX*::kan FRT+ [pST46] | This study |
| ∆serT | *E. coli* MG1655 (DE3), *∆serT*::kan FRT+ [pST51] | This study |
| ∆rrnD | *E. coli* MG1655 (DE3), *∆rrnD*::kan FRT+ | This study |
| ∆rrnH | *E. coli* MG1655 (DE3), *∆rrnH*::kan FRT+ | This study |
| ∆pheU | *E. coli* MG1655 (DE3), *∆pheU*::kan FRT+ | This study |
| ∆argU | *E. coli* MG1655 (DE3), *∆argU*::kan FRT+ [ptRNA72] | This study |
| ∆rrnC | *E. coli* MG1655 (DE3), *∆rrnC*::kan FRT+ [ptRNA73] | This study |
| ∆metY | *E. coli* MG1655 (DE3), *∆metY*::kan FRT+ | This study |
| ∆rrnE | *E. coli* MG1655 (DE3), *∆rrnE*::kan FRT+ | This study |
| ∆serW | *E. coli* MG1655 (DE3), *∆serW*::kan FRT+ | This study |
| ∆rrnG | *E. coli* MG1655 (DE3), *∆rrnG*::kan FRT+ | This study |
|  |  |  |

**Table S 6:** The table provides oligonucleotides used for the deletion of tRNA transcription units. It includes information on the targeted transcription unit genome position and the corresponding area removed from the reference genome E. coli K-12 MG1655 (EcoCyc) ^7^.

| **Targeted deletion** | **Genome map position** |  | **Sequence [5’ → 3’]** | **Area removed from the genome** |
| --- | --- | --- | --- | --- |
| *gltU-trpT-aspT (rrnC)* | 3,941,808 → 3,946,948 | Frw | TTTATTCCTCCTTAGTATGCCACCAGGAAGTGTGATTACGGTGTAGGCTGGAGCTGCTTC | 3,941,389 → 3,947,067 |
|  |  | Rev | CCGGTAGAAGGATTTACTTCGGAGAGGGTTATTTCAGATACATATGAATATCCTCCTTAG |  |
| *argX-hisR-leuT-proM* | 3,982,375 → 3,982,811 | Frw | CACGGGAAAGCGCATAAACTGGAGGAATAAGCAGCAAAACGTGTAGGCTGGAGCTGCTTC | 3,982,283 → 3,982,866 |
|  |  | Rev | CGTGGGGCAATGAGTTATTACGGGCGTTTATTGCGAAGGGCATATGAATATCCTCCTTAG |  |
| *ileT-alaT (rrnA)* | 4,035,531 → 4,040,636 | Frw | GCATAAAGAATAAAAAATGCGCGGTCAGAAAATTATTTTAGTGTAGGCTGGAGCTGCTTC | 4,035,180 → 4,040,865 |
|  |  | Rev | CAAGAGTAGGCTGGATAAGACGCGCCAGCGTCGCATCCGGCATATGAATATCCTCCTTAG |  |
| *gltT (rrnB)* | 4,166,659 → 4,171,156 | Frw | TTTGGTTGAATGTTGCGCGGTCAGAAAATTATTTTAAATTGTGTAGGCTGGAGCTGCTTC | 4,166,300 → 4,172,017 |
|  |  | Rev | AGCTGCTTTCCTGATGCAAAAACGAGGCTAGTTTACCGTACATATGAATATCCTCCTTAG |  |
| *thrU-tyrU-glyT-thrT* | 4,175,388 → 4,175,829 | Frw | TGACAGATATTCCATCTGATAAAAAGAATTATGGTTTAGCGTGTAGGCTGGAGCTGCTTC | 4,175,225 → 4,175,893 |
|  |  | Rev | CTAAGACACGGATAAATCGGTGATATCACCACATCAACCACATATGAATATCCTCCTTAG |  |
| *gltV (rrnE)* | 4,208,147 → 4,213,159 | Frw | GGTAAATCCCCTGGATTTGACTATTACAGAGAGCGTTAGCGTGTAGGCTGGAGCTGCTTC | 4,207,686 → 4,213,200 |
|  |  | Rev | TGACATGAGATTCCCTTCATCATGCAAATAATTGATATGCCATATGAATATCCTCCTTAG |  |
| *pheU* | 4,362,551← 4,362,626 | Frw | ATTGATTTGGAGGCAGAACGCTTAAATCGTGGCGTCCTGTGTAGGCTGGAGCTGCTTC | 4,363,508 ← 4,362,666 |
|  |  | Rev | CAATCTTAAGCAGTTGAATCGCTTTTACTGAAATTAGGCATATGAATATCCTCCTTAG |  |
| *glyVYX* | 4,392,360 → 4,392,658 | Frw | CTAAATTGAACGTCTTGCTGATAATTTCAGCACTTGAGATGTGTAGGCTGGAGCTGCTTC | 4,392,224 → 4,392,680 |
|  |  | Rev | ACCAGCAATAACGCATCGCGTCGCTGTGGATATTTTATTGCATATGAATATCCTCCTTAG |  |
| *leuX* | 4,496,405 → 4,496,489 | Frw | ACATCGCTGTATGCAATGCTGAAAATTTCAGCACTTAGCGAGCTGCTTCGAAGTTCCTAT | 4,496,329 → 4,496,580 |
|  |  | Rev | TGTACGTTGATATCGGTTGACCTTGAGAGAGTTAATATAGCGTCGACCTGCAGTTCGAAG |  |
| *leuQPV* | 4,606,079 ← 4,606,401 | Frw | AAAATCAAGGAAGAAACAAGAAAGGAAGTAAAGATAATTGGGCTGCTTCGAAGTTCCTAT | 4,606,082 ← 4,606,515 |
|  |  | Rev | CTGCCCCCGCATGCATACCAAACCTGCACGCTAGTTTCCTGTCGACCTGCAGTTCGAAG |  |
| *ileV-alaV-aspU (rrnH)* | 223,771 → 229,004 | Frw | TTAAAAGATGAGCGGTTGAAATAAAAATGCATTTTTCCGCGTGTAGGCTGGAGCTGCTTC | 223,449 → 229,127 |
|  |  | Rev | AATATGCCTCTTTTAATGCTATTTTCGTTAACTGAGGTGACATATGAATATCCTCCTTAG |  |
| *aspV* | 236,931 → 237,007 | Frw | CGATTTTTGCAGCAAACGATTCAAAAGATGAGAAAAACCGTTGCTGCTTCGAAGTTCCTAT | 236,976 → 237,067 |
|  |  | Rev | AGTAAAATCATTGAACAGAGAAATGGTGGAATTCAAATACATCGACCTGCAGTTCGAAG |  |
| *thrW* | 262,871 → 262,946 | Frw | TGGTGATTACACCATTCGTGCGTAAATAAAACCGGGTGATGGCTGCTTCGAAGTTCCTAT | 262,773 → 262,948 |
|  |  | Rev | GGTGGGAGAATGATAAGATCTTACGTAACAATTTGATTTTATCGACCTGCAGTTCGAAG |  |
| *argU* | 564,723 → 564,799 | Frw | CGTTTGTTTGCCGCTATAGCGAAATAAATCAGAAAATCAGGTGTAGGCTGGAGCTGCTTC | 564,624 → 564,813 |
|  |  | Rev | CCCACTCTGAAATTATGGAGGATATAAAGAAGGCGTAACTCATATGAATATCCTCCTTAG |  |
| *metT-leuW-glnW-metU-glnVX* | 697,057 ← 697,133 | Frw | GATAAGACGTGTCAACATCGCATTCGACATTGAATGAACGGTGTAGGCTGGAGCTGCTTC | 696,392 ← 697,245 |
|  |  | Rev | AAGTAAACAATAATTGACGAATATAGCGCCACGCTGTTCGCATATGAATATCCTCCTTAG |  |
| *lysT-valT-lysW-valZ-lysYZQ* | 780,554 → 781,652 | Frw | ATGCATAACACACGACCAGAAGTCGCATTATTTCTGGTCGGTGTAGGCTGGAGCTGCTTC | 780,427 → 782,031 |
|  |  | Rev | TTTACAGGCTTAACGTTAAAACCGACATTAGCGTAATATTCATATGAATATCCTCCTTAG |  |
| *serW* | 925,971 ← 925,884 | Frw | AATGATGATGGTAAGTTGTTGATAATTAGTGCTGCGGGAAGTGTAGGCTGGAGCTGCTTC | 926,082 ← 925,673 |
|  |  | Rev | CGTAACTTGTCTCGATGTAGATATACTGCACAACTTGTTTCATATGAATATCCTCCTTAG |  |
| *serT* | 1,031,625 ← 1,031,712 | Frw | TTACGCATTTGATCTGGAACAGGTTTAACAGCGGATTATCGTGTAGGCTGGAGCTGCTTC | 1,031,540 ← 1,031,818 |
|  |  | Rev | AGTAAAAGAGAAGGAATTCATGGTGATGAAATGATCAATCCATATGAATATCCTCCTTAG |  |
| *serX* | 1,097,565 ← 1,097,652 | Frw | ACCCCGGTCGGGGCTTCTCATCCCCCCGGTGTGTGCAATATGCTGCTTCGAAGTTCCTAT | 1,096,977 ← 1,097,760 |
|  |  | Rev | CGGTGGCAAAAAAAGCAGATTTCGCTTATTAAAACCACACATCGACCTGCAGTTCGAAG |  |
| *tyrTV* | 1,287,244 ← 1,287,622 | Frw | TATTTAATTGCGGATTCGTTGGGAAGTTCAGGGACTTTTGAGCTGCTTCGAAGTTCCTAT | 1,287,238 ← 1,287,741 |
|  |  | Rev | TCGCCAGCAAAAATAACTGGTTACCTTTAATCCGTTACGGATCGACCTGCAGTTCGAAG |  |
| *valVW* | 1,746,435 → 1,746,592 | Frw | TGTAAATGCGATCCTGAATAAAAATCACCCTTGCAAATCAAGCTGCTTCGAAGTTCCTAT | 1,746,309 → 1,746,624 |
|  |  | Rev | TGTAAATCTTTTAACGTATTGGATACAGAGAAGAAACAGAGTCGACCTGCAGTTCGAAG |  |
| *glyW-cysT-leuZ* | 1,991,815 ← 1,992,117 | Frw | CAGGCACAAAAAAACCACCCGAAGGTGGTTTCACGAACGTCTTGAGCGATTGTGTAG | 1,991,777 ← 1,992,222 |
|  |  | Rev | CGTAATTTTCAGCAAACGATCAAAAGTGGTGAAAAATACATATGAATATCCTCCTTAG |  |
| *serU* | 2,043,468 ←  2,043,557 | Frw | AACCCTTCAAGAATATTCTACGATTGTTCTGTTTAGGAAAAGCTGCTTCGAAGTTCCTAT | 2,043,622 ← 2,043,431 |
|  |  | Rev | GCCACTTAATCATCGTTTTGCTCGCAAACTCGTCACTTGAACTCGACCTGCAGTTCGAAG |  |
| *asnT* | 2,044,549 → 2,044,624 | Frw | ACAACTTTGCAGATTAATTAACCAATTGAAATGACTTATGGTGTAGGCTGGAGCTGCTTC | 2,044,492 → 2,044,752 |
|  |  | Rev | TAACTCAAGTTTTATAATCGAGGGGAAAATGGTGATGGCGCATATGAATATCCTCCTTAG |  |
| *asnW* | 2,058,127 ← 2,058,102 | Frw | GACAATTTGCTCACTGAAGCGTGAGACTCGATTAAGCGCACGCTGCTTCGAAGTTCCTAT | 2,057,916 ← 2,058,133 |
|  |  | Rev | GATTTCAAATTTAAAAAACAGGCTTTGACATTGTGGGTGGGTCGACCTGCAGTTCGAAG |  |
| *asnU* | 2,059,851 → 2,059,926 | Frw | CAAATTGTCTGCAAATGCAACAAACTGTTGATAGAAACGGGTGTAGGCTGGAGCTGCTTC | 2,059,778 → 2,059,950 |
|  |  | Rev | AAGAGGAAATTGATTATCAGATATAGCAAAAATCCCGCTACATATGAATATCCTCCTTAG |  |
| *asnV* | 2,062,260 → 2,062,335 | Frw | TTGAAAGCTCACCACTTTGTTGAAATTGACAGCAAACAAACGCTGCTTCGAAGTTCCTAT | 2,062,193 → 2,062,385 |
|  |  | Rev | GTGCAAATTAGTTCTGGTTCAAGACAGATGTTTTAAGAGCGTTCGACCTGCAGTTCGAAG |  |
| *proL* | 2,286,211 → 2,286,287 | Frw | GCAAGTGCTGACAGACGAGAAGCGTTTTATCGCTAACTGAGCTGCTTCGAAGTTCCTAT | 2,286,100 → 2,286,337 |
|  |  | Rev | AACAGGCTGTGATGTGTCACGGTCTGTTTATCGAATTAATTTCGACCTGCAGTTCGAAG |  |
| *argW* | 2,466,309 → 2,466,383 | Frw | AGAACGTGCGGAAAACATTAAGAAAAATTATAAAAACCCGGGCTGCTTCGAAGTTCCTAT | 2,466,229 → 2,466,387 |
|  |  | Rev | CAGGGGATTTTGCGGACTGGTACGGATGGGAGCGAACTGATTCGACCTGCAGTTCGAAG |  |
| *alaWX* | 2,518,116 ← 2,518,231 | Frw | ACCCGGACGTAGACTGTATTACAAAAGCGGCAAAAAGCAGGTGTAGGCTGGAGCTGCTTC | 2,518,002 ← 2,518,356 |
|  |  | Rev | ACAGGAAACCGTGACATGAATAACTACTGAATGCGAGCGGCATATGAATATCCTCCTTAG |  |
| *valUXY-lysV* | 2,520,931 → 2,521,328 | Frw | ACCGGCAAATCCTGGAAAAATTAAGTGGGCGATATTACGTCTTGAGCGATTGTGTAG | 2,520,710 → 2,521,501 |
|  |  | Rev | ATTTCGCAAATCGCAGATAGCAAAAAAGCGCCTTTAGATCAGTGATAAGCTGTCAAAC |  |
| *gltW (rrnG)* | 2,726,069 ← 2,731,157 | Frw | GCCATTATGTCTCCTGCCGTAATCCGATGCTTTTGTCGGTGTGTAGGCTGGAGCTGCTTC | 2,725,787 ← 2,731,560 |
|  |  | Rev | ATGATAAAACGAGCCCTTCGGGGCTCGTTTTTGTCTATAACATATGAATATCCTCCTTAG |  |
| *ileY* | 2,785,762 ← 2,785,837 | Frw | CTTGAAATGGCGTTAGTCATGAAATATAGACCGCCATCGAGTGTAGGCTGGAGCTGCTTC | 2,785,717 ← 2,785,919 |
|  |  | Rev | GGTTTTATGTCGCGTGTAATCACGACCCAAGGTGAATGGGCATATGAATATCCTCCTTAG |  |
| *argQZYV-serV* | 2,817,784 ← 2,818,645 | Frw | TATTTTTGAAGGAAATTTTTTGGAGAAGATGGTGCATCCGGTGTAGGCTGGAGCTGCTTC | 2,817,662 ← 2,818,794 |
|  |  | Rev | AGACCTCCATTCTTCTGTTGATAAAACACTCTTTTTGACGCATATGAATATCCTCCTTAG |  |
| *metZWV* | 2,947,387 → 2,947,683 | Frw | TTTGTTCAAAATCATGCCAAATCCGTGATCGGGGTAAAAAAGCTGCTTCGAAGTTCCTAT | 2,947,342 → 2,947,729 |
|  |  | Rev | GAGAAGGGGATGATAAAAAGGCGCTGAATGGCGCTTTTTTATCGACCTGCAGTTCGAAG |  |
| *glyU* | 2,998,984 ← 2,999,057 | Frw | CTAAATTGAACGTCTTGCTGATAATTTCAGCACTTGAGATGTGTAGGCTGGAGCTGCTTC | 2,998,966 ← 2,999,136 |
|  |  | Rev | ACCAGCAATAACGCATCGCGTCGCTGTGGATATTTTATTGCATATGAATATCCTCCTTAG |  |
| *pheV* | 3,110,366 → 3,110,441 | Frw | GTGATGATAGATTGTGCAGTCTGCAGTAAATTGAAGGTGTAGGCTGGAGCTGCTTC | 3,110,319 → 3,110,455 |
|  |  | Rev | CAGATCCAAAAGCAAAAACCCGCCTTGTGGGCGGGTCATATGAATATCCTCCTTAG |  |
| *ileX* | 3,215,598 → 3,215,673 | Frw | AAATACATTGTTTATAAAAACAGCAGGCGCGCGGTAATGGGTGTAGGCTGGAGCTGCTTC | 3,215,558 → 3,215,717 |
|  |  | Rev | CGTGCAGCGGCTTACTGGCACGCAAAGTAACTGATATATACATATGAATATCCTCCTTAG |  |
| *metY* | 3,218,213 ← 3,318,289 | Frw | TCTTCACAGTATATTTGAAAAAGGACTCTAAGGGAAAGGTGTAGGCTGGAGCTGCTTC | 3,318,213 ← 3,318,363 |
|  |  | Rev | TGTGGCGTGGATCACTATAATGCCTGCAGATTTTACGTCCCATATGAATATCCTCCTTAG |  |
| *leuU* | 3,322,072 ← 3,322,158 | Frw | CGTTTCAAGGTACCAGCTACGAGTAAAGCAACTGGACGAGGTGTAGGCTGGAGCTGCTTC | 3,321,993 ← 3,322,173 |
|  |  | Rev | AGCTGCTCCGGCTAAGCCGACCAGCGATATCCCGAACTAACATATGAATATCCTCCTTAG |  |
| *ileU-alaU-thrV (rrnD)* | 3,423,423 ← 3,428,762 | Frw | TTGAATGTTAGTTCGAAAAGCAAAAAGGCCATCCTTTCGGGTGTAGGCTGGAGCTGCTTC | 3,423,235 ← 3,429,196 |
|  |  | Rev | ACTTACCTCAGCAATAAATGATTTACTAATGACTTTGGGGCATATGAATATCCTCCTTAG |  |
| *proK* | 3,708,616 ← 3,708,692 | Frw | TCGTGGCTCGCCCTGCGGGCCGTTGCTGGCGCAACGTTCTCGCTGCTTCGAAGTTCCTAT | 3,708,518 ← 3,708,784 |
|  |  | Rev | GGTGTCACATCCCGCAGGCAAAAGAGGCAGCGGCTAACTAATCGACCTGCAGTTCGAAG |  |
| *selC* | 3,836,222 → 3,836,316 | Frw | GTTTTATATGAACTATAATGCTTTCGTGATAATACGCTGCGTGTAGGCTGGAGCTGCTTC | 3,836,131 → 3,836,356 |
|  |  | Rev | GCCGATTGAATATTGAGCAGTAGAAAATATCTGGATTGACCATATGAATATCCTCCTTAG |  |

**Table S 7:** Oligonucleotides used for the cPCR verification of tRNA transcription unit removal.

| **Targeted gene deletion** |  | **Sequence [5’→ 3’]** |
| --- | --- | --- |
|  |  |  |
| *gltU-trpT-aspT (rrnC)* | Frw | GCAGCTAACTGTTGTGCGCTTA |
|  | Rev | ATGCGTTGATTCGCGATCTTTTG |
| *argX-hisR-leuT-proM* | Frw | GAAAGCGCATAAACTGGAGGAATAAG |
|  | Rev | GGTTCGCCGCCCTGCCAGGTAAAAG |
| *ileT-alaT (rrnA)* | Frw | CCATTTAACCGACAAACCGACGCTG |
|  | Rev | TGTGGTGGAGTGGATGCAAAAGCAG |
| *gltT (rrnB)* | Frw | CAGCGTTACGGCTTCGAAACG |
|  | Rev | GATCAATGCCAAATGTGTTCCAG |
| *thrU-tyrU-glyT-thrT* | Frw | GTCATAGGTACGGAATCACGCAGAGC |
|  | Rev | GGTCAACGTGGCCGATAGTACCGACG |
| *gltV (rrnE)* | Frw | GTCAGAAACACTGAGCAGAGCGC |
|  | Rev | GATTACGACGAATTTGGATATAC |
| *pheU* | Frw | GCTCCTGATGACGTCATTTGACG |
|  | Rev | CATCGGCTGGCGGAAGATATC |
| *glyVYX* | Frw | GACGCATCAGGCGATGGATGATATCC |
|  | Rev | CGTGCATAGTCGAAGTGCAATTGCCG |
| *leuX* | Frw | CGTACTCATAAACTTCCAGTTCGCCG |
|  | Rev | GCTTATCCGTTGGCTTGGCTGCTCTG |
| *leuQPV* | Frw | GCTACCATCGTGCCTGGCACTTATTG |
|  | Rev | CCTTATCCGGACGTGCTGGATGAGAC |
| *ileV-alaV-aspU (rrnH)* | Frw | CAGACCTGCCGCAAGCGATA |
|  | Rev | GCGCCGTTATCACAGATGAAATAAC |
| *aspV* | Frw | GGAGAGACACAACAGCAACAAGGTG |
|  | Rev | CAATAGAAGATTCAGAGGCACATCCTGC |
| *thrW* | Frw | GTCTGGGTGCGGAAGTGGCGGTAAGC |
|  | Rev | GCTGCAGACATTCTGCGAGCGCGGC |
| *argU* | Frw | GCAACAGAACTTCAATAGACATTCTGATG |
|  | Rev | TAGGTATGCTCACCTTGCGCCTAATC |
| *metT-leuW-glnUW-metU-glnVX* | Frw | GTAGGTCGGATAAGACGCGGCAG |
|  | Rev | TCCTGTATGACAAAAGAATGGCTGTAGG |
| *lysT-valT-lysW-valZ-lysYZQ* | Frw | GAAAGCCGTATACCAGCAGGTTATC |
|  | Rev | CATCAATGCTTAACGGCGTCGGCTTC |
| *serW* | Frw | GAGCTGACTTACGGATTCCAGAGTGC |
|  | Rev | CCTGACGGGCGACAAAGTGACTGTTG |
| *serT* | Frw | GCGTCAGAATCAGACCCGGAGATGGC |
|  | Rev | CCCAGCGACGTGGCAGCCAGACTAC |
| *serX* | Frw | GACTGAGTAACCAGACAGTTGATGTG |
|  | Rev | AAGATGATATCCATACTGACTCCCG |
| *tyrTV* | Frw | GAGTATCTGACACGTAAGTACCAGC |
|  | Rev | CCTACACAGCTGAAGATATGATGCGC |
| *valVW* | Frw | CTCATCAAGTTTTTCGCATCAGATCC |
|  | Rev | GTAACAAAGTTGCCATAGCGATTCTC |
| *glyW-cysT-leuZ* | Frw | CTCAACTCGCCAGCACCTATC |
|  | Rev | CGGCGTAATTTTCAGCAAACG |
| *serU* | Frw | CTACGCAACTTCAGTTCCCACCAC |
|  | Rev | GCTTTGTTCCTGTTCCGTCAGACACG |
| *asnT* | Frw | GATGCTTATGCAGCCAGCGATCCTGC |
|  | Rev | CAGATACCCGCAGTTAAGCGGCGTAG |
| *asnW* | Frw | GGCTTGTTGATGCCCTTGAAGATGAC |
|  | Rev | GAATGTTTGCCGACTGGGCTGTGCG |
| *asnU* | Frw | GTGAACAACCTGGCGTAAAGCAGAGG |
|  | Rev | CAACTTCAGCGTAACTATGTCTGGCG |
| *asnV* | Frw | GCAATACTTCAGCAGCCTGGGTCAG |
|  | Rev | CGGGCAAGAAGTTGATGTAAATCGCG |
| *proL* | Frw | CCGCACTTACAACTTACGTGGTGAAAG |
|  | Rev | GTAGAATCGCCGTGGAATACTCAGGC |
| *argW* | Frw | CATCTGCGGCGGCACCTTTATCTTCG |
|  | Rev | CAGGCCATTACCATCGAGAAGGCGG |
| *alaWX* | Frw | GTACCACGGTGAGCGTAAACGCTAGTG |
|  | Rev | CGTTGTCCAGCCTTCCTTGCGTACCC |
| *valUXY-lysV* | Frw | GTCTTCAATACGCAGCACGAACTC |
|  | Rev | GTATCACATTGATAGCGGACAGCTAC |
| *gltW (rrnG)* | Frw | CGGCGACGAGTATCACTACTTGTC |
|  | Rev | TCGCCTGGAAGTTAATGAAGACC |
| *ileY* | Frw | CTGCGCTAGCGTTAAATCCGTTGC |
|  | Rev | GTCGATAGGTAAATTTCTGGGTCCTG |
| *argQZYV-serV* | Frw | GTAGGCTCCGTATCCAGGATTGTGCC |
|  | Rev | GAGATCTACCAGCGTATCCAGGCTG |
| *metZWV* | Frw | GCGGCAAGCATTGCCACAACCGTGC |
|  | Rev | CGTATTTTGCCGATGGGGCGACGCTGG |
| *glyU* | Frw | TACAGATTCACTCGTCACTTCTGCCC |
|  | Rev | CTTACGCCCATAATGACACGATGCTGG |
| *pheV* | Frw | ACCGCCTGGTTTGCCTG |
|  | Rev | GGCGAGAGTAATTCCCCC |
| *ileX* | Frw | CGGCGGATGAAAGCCCAGGGTTGATAC |
|  | Rev | AACAGTATGACCCACAGCGGGTACGT |
| *metY* | Frw | GCACAGCACTTACCTGGTGGCTCAC |
|  | Rev | TCCCTTTTGTCGCATCCACAGCAGTG |
| *leuU* | Frw | CGCAGAACGTAGCCATCAGTCGTTATAC |
|  | Rev | CTTCATCATCAGTCTGGTGCTGGGTAAC |
| *ileU-alaU-thrV (rrnD)* | Frw | TCAGAGCGTACGAGGGCATT |
|  | Rev | ATTACGCGCTGACCGATTTGTGG |
| *proK* | Frw | GCGCAAATTTGGCAGGGAAATGTGTG |
|  | Rev | CCGTCACGTAGAGCTGTACGATACCATC |
| *selC* | Frw | GGACAACACTTCACTCTTC |
|  | Rev | GATTCTTCAGCCGCTCATC |
|  |  |  |

**Table S 8:** DNA sequences obtained by DNA synthesis containing *argU* and *rrnC* tRNA genes (*aspT, trpT* and *gltT*) under control of *lpp* promoter.

| ACACCGAGTGTGGATCTGCCCCATCAAAAAAATATTCTCAACATAAAAAACTTTGTGTAATACTTGTAACGCTGCGCCCTTAGCTCAGTTGGATAGAGCAACGACCTTCTAAGTCGTGGGCCGCAGGTTCGAATCCTGCAGGGCGCGCCAATCCTTAGCGAAAGCTAAGGATTTTTTTTCCTGACTGCTCAAGAACTTA |
| --- |
| GTATTAGTCGTCCGTAGTGCCCATCAAAAAAATATTCTCAACATAAAAAACTTTGTGTAATACTTGTAACGCTGGAGCGGTAGTTCAGTCGGTTAGAATACCTGCCTGTCACGCAGGGGGTCGCGGGTTCGAGTCCCGTCCGTTCCGCCACCCTAATTAGGGGCGTAGTTCAATTGGTAGAGCACCGGTCTCCAAAACCGGGTGTTGGGAGTTCGAGTCTCTCCGCCCCTGCCATCTCTGTAGTGATTAAGAGCGTGATAAGCAATTTTCGTGTCCCCTTCGTCTAGAGGCCCAGGACACCGCCCTTTCACGGCGGTAACAGGGGTTCGAATCCCCTAGGGGACGCCAATCCTTAGCGAAAGCTAAGGATTTTTTTTTTCGTGCCGCGCAAGCAC |

**Table S 9:** Oligonucleotides used for complementing tRNA plasmid (ptRNA) assembly. *tRNA transcription unit obtained through DNA synthesis. The other transcription units were amplified using PCR with genomic DNA as the template.

| **Trageted amplification** |  | **Sequence [5’ → 3’]** |
| --- | --- | --- |
| pSC101 plasmid backbone | Frw | CATGCAAGCTCTAGAGGCATC |
|  | Rev | GTCGGAATTGCCAGCTGGGG |
| *rrnC** | Frw | CAAAGGATCTTCTTGAGATCCCCAGCTGGCAATTCCGACCTGTATTAGTCGTCCGTAGTG |
|  | Rev | ACTGAGCCTTTCGTTTTATTTGATGCCTCTAGAGCTTGCATGGTGCTTGCGCGGCACGAA |
| *argX-hisR-leuT-proM* | Frw | CAAAGGATCTTCTTGAGATCCCCAGCTGGCAATTCCGACCGCACAAACCGTAACCAAACG |
|  | Rev | TCGTTTTATTTGATGCCTCTAGAGCTTGCATGGTGACGAAATGCACAGAAAAC |
| *thrU-tyrU-glyT-thrT* | Frw | GGATCTTCTTGAGATCCCCAGCTGGCAATTCCGACGGAGCGCATTGTTGAGCAC |
|  | Rev | TGAGCCTTTCGTTTTATTTGATGCCTCTAGAGCTTGCATGGCAACATGCCCGACTTGTTG |
| *argU** | Frw | GATCTTCTTGAGATCCCCAGCTGGCAATTCCGACGACGCGGTCGTTCACTTGTTCAG |
|  | Rev | TTTCGTTTTATTTGATGCCTCTAGAGCTTGCATGGATTGAATTGTAATGGCGCGCC |
| *metT-leuW-glnUW-metU- glnVX* | Frw | AAAGGATCTTCTTGAGATCCCCAGCTGGCAATTCCGACGAATGAACGCAGAAAAGCAAAA |
|  | Rev | TGAGCCTTTCGTTTTATTTGATGCCTCTAGAGCTTGCATGACCAAACAGTCACTTTCGAG |
| *lysT-valT-lysW-valZ-lysYZQ* | Frw | TCAAAGGATCTTCTTGAGATCCCCAGCTGGCAATTCCGACGTCGTGCGAATCATAAGCAG |
|  | Rev | TGAGCCTTTCGTTTTATTTGATGCCTCTAGAGCTTGCATGATTGAGGATGCCTGAATAGC |
| *serT* | Frw | CCCCAGCTGGCAATTCCGACCATTAAGCAAATATAACGCCCTG |
|  | Rev | ATGCCTCTAGAGCTTGCATGCGCTATCAATGCACTTTTGGCTG |
| *glyW-cysT-leuZ* | Frw | GGATCTTCTTGAGATCCCCAGCTGGCAATTCCGACGTGTAACGACAAGTTGCAGGC |
|  | Rev | GAGCCTTTCGTTTTATTTGATGCCTCTAGAGCTTGCATGGTTGACTCATCGCGCCAGGT |
| *argQZYV-serV* | Frw | ACTTTTATCGTTTGCACACTTCACGTTCAATTAGTCTGTCATGCAAGCTCTAGAGGCATC |
|  | Rev | CGGCCCGAAGGGCGAACGTCAGTGAGTCATCCTCGTCGGAATTGCCAGCTGGGG |
| *ileX* | Frw | AAGGATCTTCTTGAGATCCCCAGCTGGCAATTCCGACGCTGGATTGCGACACGGAGTTAC |
|  | Rev | TTTTATTTGATGCCTCTAGAGCTTGCATGAAAAAGAAAAAGGCTGACGATTTCTCG |

**Supplementary sequences of selected plasmids**

pST40

*GTCGTGCGAATCATAAGCAGTTGAGTGATCTACATCGAAATTTTTGTTGCGCTCAAGTCTGAAATCAGTAATATATGCCGCCGTTGCCACGGGATATCAAACAAACCGAAAGCAACGAAAAAGTGGGTCGTTAGCTCAGTTGGTAGAGCAGTTGACTTTTAATCAATTGGTCGCAGGTTCGAATCCTGCACGACCCACCAATCGCTAAGGTGGAAGCGGTAGTAAAACGTGAAGGATAACGTTGCATGAGCAACGGCCCGAAGGGCGAGACGAAGTCGAGTCATCCTGCACGACCCACCACTAACATAGTTAGTTGTAGTATCCAGCGTAGTATCGGGTGATTAGCTCAGCTGGGAGAGCACCTCCCTTACAAGGAGGGGGTCGGCGGTTCGATCCCGTCATCACCCACCACCGGGTCGTTAGCTCAGTTGGTAGAGCAGTTGACTTTTAATCAATTGGTCGCAGGTTCGAATCCTGCACGACCCACCAGTTTTAACATCGAAGACAGATGTTAAGCGTGTAGGATAACGTTGCGTCAGCAACGGCCCGTAGGGCGAGCGAAGCGAGTCATCCTGCACGACCCACCACTAATGACGGTGGGTTCGGTGGAAGTAGTTTGTAGTATCCAGCGCAGTATCGGGTGATTAGCTCAGCTGGGAGAGCACCTCCCTTACAAGGAGGGGGTCGGCGGTTCGATCCCGTCATCACCCACCACTCGGGTCGTTAGCTCAGTTGGTAGAGCAGTTGACTTTTAATCAATTGGTCGCAGGTTCGAATCCTGCACGACCCACCAGTTTTAACATCAAACTCAGATGTTAAGCGTGAAGGATAACGTTGCGCCAGCAACGGCCCGTAGGGCGAGCGAAGCGAGTCATCCTGCACGACCCACCAATCTTAAAGATTGGCCCCGAGTAAAAATCTTTCAGGTAACACCCGTATGGGTCGTTAGCTCAGTTGGTAGAGCAGTTGACTTTTAATCAATTGGTCGCAGGTTCGAATCCTGCACGACCCACCAATTTAAAGGTGGTTACTGGTAGAGAACGTGAAGGATAACGTTGCGTTAGCAACGGCCCGAAGGGCGAGACGAAGTCGAGTCATCCTGCACGACCCACCATCCTGAATGATTAAGGCAGCATAATCCCGCAAGGGGTCGTTAGCTCAGTTGGTAGAGCAGTTGACTTTTAATCAATTGGTCGCAGGTTCGAATCCTGCACGACCCACCAATGTAAAAAAGCGCCCTAAAGGCGCTTTTTTGCTATCTGCGATACTCAAAGATTCGAACCTGCAGCAGGTTTGAGTTGAGCGCAGCGAAACAACGGAGCCGCTCGCGGCGACGGCCCGAAGGGCGAGCGAAGCGAGTCATCCTGCACGACCCACCAATGTAAAAAAGCGCCCTAAAGGCGCTTTTTTGCTATTCAGGCATCCTCAATCATGCAAGCTCTAGAGGCATCAAATAAAACGAAAGGCTCAGTCGAAAGACTGGGCCTTTCGTTTTATCTGTTGTTTGTCGGTGAACGCTCTCCTGAGTAGGACAAATCCGCCGCCCTAGACCTAGGGTACGGGTTTTGCTGCCCGCAAACGGGCTGTTCTGGTGTTGCTAGTTTGTTATCAGAATCGCAGATCCGGCTTCAGCCGGTTTGCCGGCTGAAAGCGCTATTTCTTCCAGAATTGCCATGATTTTTTCCCCACGGGAGGCGTCACTGGCTCCCGTGTTGTCGGCAGCTTTGATTCGATAAGCAGCATCGCCTGTTTCAGGCTGTCTATGTGTGACTGTTGAGCTGTAACAAGTTGTCTCAGGTGTTCAATTTCATGTTCTAGTTGCTTTGTTTTACTGGTTTCACCTGTTCTATTAGGTGTTACATGCTGTTCATCTGTTACATTGTCGATCTGTTCATGGTGAACAGCTTTGAATGCACCAAAAACTCGTAAAAGCTCTGATGTATCTATCTTTTTTACACCGTTTTCATCTGTGCATATGGACAGTTTTCCCTTTGATATGTAACGGTGAACAGTTGTTCTACTTTTGTTTGTTAGTCTTGATGCTTCACTGATAGATACAAGAGCCATAAGAACCTCAGATCCTTCCGTATTTAGCCAGTATGTTCTCTAGTGTGGTTCGTTGTTTTTGCGTGAGCCATGAGAACGAACCATTGAGATCATACTTACTTTGCATGTCACTCAAAAATTTTGCCTCAAAACTGGTGAGCTGAATTTTTGCAGTTAAAGCATCGTGTAGTGTTTTTCTTAGTCCGTTATGTAGGTAGGAATCTGATGTAATGGTTGTTGGTATTTTGTCACCATTCATTTTTATCTGGTTGTTCTCAAGTTCGGTTACGAGATCCATTTGTCTATCTAGTTCAACTTGGAAAATCAACGTATCAGTCGGGCGGCCTCGCTTATCAACCACCAATTTCATATTGCTGTAAGTGTTTAAATCTTTACTTATTGGTTTCAAAACCCATTGGTTAAGCCTTTTAAACTCATGGTAGTTATTTTCAAGCATTAACATGAACTTAAATTCATCAAGGCTAATCTCTATATTTGCCTTGTGAGTTTTCTTTTGTGTTAGTTCTTTTAATAACCACTCATAAATCCTCATAGAGTATTTGTTTTCAAAAGACTTAACATGTTCCAGATTATATTTTATGAATTTTTTTAACTGGAAAAGATAAGGCAATATCTCTTCACTAAAAACTAATTCTAATTTTTCGCTTGAGAACTTGGCATAGTTTGTCCACTGGAAAATCTCAAAGCCTTTAACCAAAGGATTCCTGATTTCCACAGTTCTCGTCATCAGCTCTCTGGTTGCTTTAGCTAATACACCATAAGCATTTTCCCTACTGATGTTCATCATCTGAGCGTATTGGTTATAAGTGAACGATACCGTCCGTTCTTTCCTTGTAGGGTTTTCAATCGTGGGGTTGAGTAGTGCCACACAGCATAAAATTAGCTTGGTTTCATGCTCCGTTAAGTCATAGCGACTAATCGCTAGTTCATTTGCTTTGAAAACAACTAATTCAGACATACATCTCAATTGGTCTAGGTGATTTTAATCACTATACCAATTGAGATGGGCTAGTCAATGATAATTACTAGTCCTTTTCCTTTGAGTTGTGGGTATCTGTAAATTCTGCTAGACCTTTGCTGGAAAACTTGTAAATTCTGCTAGACCCTCTGTAAATTCCGCTAGACCTTTGTGTGTTTTTTTTGTTTATATTCAAGTGGTTATAATTTATAGAATAAAGAAAGAATAAAAAAAGATAAAAAGAATAGATCCCAGCCCTGTGTATAACTCACTACTTTAGTCAGTTCCGCAGTATTACAAAAGGATGTCGCAAACGCTGTTTGCTCCTCTACAAAACAGACCTTAAAACCCTAAAGGCTTAAGTAGCGCCGTCGCAAGCTCCGGCAAATCGCTGAATATTCCTTTTGTCTCCGACCATCAGGCACCTGAGTCGCTGTCTTTTTCGTCACATTCAGTTCGCTGCGCTCACGGCTCTGGCAGTGAATGGGGGTAAATGGCACTACAGGCGCCTTTTATGGATTCATGCAAGGAAACTACCCATAATACAAGAAAAGCCCGTCACGGGCTTCTCAGGGCGTTTTATGGCGGGTCTGCTATGTGGTGCTATCTGACTTTTTGCTGTTCAGCAGTTCCTGCCCTCTGATTTTCCAGTCTGACCACTTCGGATTATCCCGTGACAGGTCATTCAGACTGGCTAATGCACCCAGTAAGGCAGCGGTATCATCAACAGGCTTACCCGTCTTACTGTCCCTAGTGCTTGGATTCTCACCAATAAAAAACGCCCGGCGGCAACCGAGCGTTCTGAACAAATCCAGATGGAGTTCTGAGGTCATTACTGGATCTATCAACAGGAGTCCAAGCGAGCTCTCGAACCCCAGAGTCCCGCTATGTATCCGCTCATGAATTAATTCTTATTACGCCCCGCCCTGCCACTCATCGCAGTACTGTTGTAATTCATTAAGCATTCTGCCGACATGGAAGCCATCACAAACGGCATGATGAACCTGAATCGCCAGCGGCATCAGCACCTTGTCGCCTTGCGTATAATATTTGCCCATGGTGAAAACGGGGGCGAAGAAGTTGTCCATATTGGCCACGTTTAAATCAAAACTGGTGAAACTCACCCAGGGATTGGCTGAGACGAAAAACATATTCTCAATAAACCCTTTAGGGAAATAGGCCAGGTTTTCACCGTAACACGCCACATCTTGCGAATATATGTGTAGAAACTGCCGGAAATCGTCGTGGTATTCACTCCAGAGCGATGAAAACGTTTCAGTTTGCTCATGGAAAACGGTGTAACAAGGGTGAACACTATCCCATATCACCAGCTCACCGTCTTTCATTGCCATACGAAATTCCGGATGAGCATTCATCAGGCGGGCAAGAATGTGAATAAAGGCCGGATAAAACTTGTGCTTATTTTTCTTTACGGTCTTTAAAAAGGCCGTAATATCCAGCTGAACGGTCTGGTTATAGGTACATTGAGCAACTGACTGAAATGCCTCAAAATGTTCTTTACGATGCCATTGGGATATATCAACGGTGGTATATCCAGTGATTTTTTTCTCCATTTTAGCTTCCTTAGCTCCTGAAAATCTCGATAACTCAAAAAATACGCCCGGTAGTGATCTTATTTCATTATGGTGAAAGTTGGAACCTCTTACGTGCCGATCAAGATCAAAGGATCTTCTTGAGATCCCCAGCTGGCAATTCCGAC*

pST41 *GAATGAACGCAGAAAAGCAAAAAGCTCGCCGAAGCGAGCTTTTTTAATGTGGCTGGGGTACGAGGATTCGAACCTCGGAATGCCGGAATCAGAATCCGGTGCCTTACCGCTTGGCGATACCCCAACTGGGTGCACTTACAAGGTAAGCGTCTTGAATAAATTGGCTGGGGTACGAGGATTCGAACCTCGGAATGCCGGAATCAGAATCCGGTGCCTTACCGCTTGGCGATACCCCAACAAATTGGTTTTGAATTTGCCGAACATATTCGATACATTCAGAATTTGGTGGCTACGACGGGATTCGAACCTGTGACCCCATCATTATGAGTGATGTGCTCTAACCAACTGAGCTACGTAGCCAGATTGTTTCTTCGATGGCTGGGGTACCTGGATTCGAACCAGGGAATGCCGGTATCAAAAACCGGTGCCTTACCGCTTGGCGATACCCCAATAACCGGGCGGTGAACCGCTTACTCGAAGAAGATGGCTGGGGTACCTGGATTCGAACCAGGGAATGCCGGTATCAAAAACCGGTGCCTTACCGCTTGGCGATACCCCATCCGTACAACGCTTTCTGGTGAATGGTGCGGGAGGCGAGACTTGAACTCGCACACCTTGCGGCGCCAGAACCTAAATCTGGTGCGTCTACCAATTTCGCCACTCCCGCAAAAAAAGATGGTGGCTACGACGGGATTCGAACCTGTGACCCCATCATTATGAGTGATGTGCTCTAACCAACTGAGCTACGTAGCCATCTTTTTTTTCGCGATACCTTATCGGCGTTGCGGGGCGCATTATGCGTATAGAGCCTTGCAGCGTCAACCTCTTTTTCAAGGAAAATTGCTCGAAAGTGACTGTTTGGTCATGCAAGCTCTAGAGGCATCAAATAAAACGAAAGGCTCAGTCGAAAGACTGGGCCTTTCGTTTTATCTGTTGTTTGTCGGTGAACGCTCTCCTGAGTAGGACAAATCCGCCGCCCTAGACCTAGGGTACGGGTTTTGCTGCCCGCAAACGGGCTGTTCTGGTGTTGCTAGTTTGTTATCAGAATCGCAGATCCGGCTTCAGCCGGTTTGCCGGCTGAAAGCGCTATTTCTTCCAGAATTGCCATGATTTTTTCCCCACGGGAGGCGTCACTGGCTCCCGTGTTGTCGGCAGCTTTGATTCGATAAGCAGCATCGCCTGTTTCAGGCTGTCTATGTGTGACTGTTGAGCTGTAACAAGTTGTCTCAGGTGTTCAATTTCATGTTCTAGTTGCTTTGTTTTACTGGTTTCACCTGTTCTATTAGGTGTTACATGCTGTTCATCTGTTACATTGTCGATCTGTTCATGGTGAACAGCTTTGAATGCACCAAAAACTCGTAAAAGCTCTGATGTATCTATCTTTTTTACACCGTTTTCATCTGTGCATATGGACAGTTTTCCCTTTGATATGTAACGGTGAACAGTTGTTCTACTTTTGTTTGTTAGTCTTGATGCTTCACTGATAGATACAAGAGCCATAAGAACCTCAGATCCTTCCGTATTTAGCCAGTATGTTCTCTAGTGTGGTTCGTTGTTTTTGCGTGAGCCATGAGAACGAACCATTGAGATCATACTTACTTTGCATGTCACTCAAAAATTTTGCCTCAAAACTGGTGAGCTGAATTTTTGCAGTTAAAGCATCGTGTAGTGTTTTTCTTAGTCCGTTATGTAGGTAGGAATCTGATGTAATGGTTGTTGGTATTTTGTCACCATTCATTTTTATCTGGTTGTTCTCAAGTTCGGTTACGAGATCCATTTGTCTATCTAGTTCAACTTGGAAAATCAACGTATCAGTCGGGCGGCCTCGCTTATCAACCACCAATTTCATATTGCTGTAAGTGTTTAAATCTTTACTTATTGGTTTCAAAACCCATTGGTTAAGCCTTTTAAACTCATGGTAGTTATTTTCAAGCATTAACATGAACTTAAATTCATCAAGGCTAATCTCTATATTTGCCTTGTGAGTTTTCTTTTGTGTTAGTTCTTTTAATAACCACTCATAAATCCTCATAGAGTATTTGTTTTCAAAAGACTTAACATGTTCCAGATTATATTTTATGAATTTTTTTAACTGGAAAAGATAAGGCAATATCTCTTCACTAAAAACTAATTCTAATTTTTCGCTTGAGAACTTGGCATAGTTTGTCCACTGGAAAATCTCAAAGCCTTTAACCAAAGGATTCCTGATTTCCACAGTTCTCGTCATCAGCTCTCTGGTTGCTTTAGCTAATACACCATAAGCATTTTCCCTACTGATGTTCATCATCTGAGCGTATTGGTTATAAGTGAACGATACCGTCCGTTCTTTCCTTGTAGGGTTTTCAATCGTGGGGTTGAGTAGTGCCACACAGCATAAAATTAGCTTGGTTTCATGCTCCGTTAAGTCATAGCGACTAATCGCTAGTTCATTTGCTTTGAAAACAACTAATTCAGACATACATCTCAATTGGTCTAGGTGATTTTAATCACTATACCAATTGAGATGGGCTAGTCAATGATAATTACTAGTCCTTTTCCTTTGAGTTGTGGGTATCTGTAAATTCTGCTAGACCTTTGCTGGAAAACTTGTAAATTCTGCTAGACCCTCTGTAAATTCCGCTAGACCTTTGTGTGTTTTTTTTGTTTATATTCAAGTGGTTATAATTTATAGAATAAAGAAAGAATAAAAAAAGATAAAAAGAATAGATCCCAGCCCTGTGTATAACTCACTACTTTAGTCAGTTCCGCAGTATTACAAAAGGATGTCGCAAACGCTGTTTGCTCCTCTACAAAACAGACCTTAAAACCCTAAAGGCTTAAGTAGCGCCGTCGCAAGCTCCGGCAAATCGCTGAATATTCCTTTTGTCTCCGACCATCAGGCACCTGAGTCGCTGTCTTTTTCGTCACATTCAGTTCGCTGCGCTCACGGCTCTGGCAGTGAATGGGGGTAAATGGCACTACAGGCGCCTTTTATGGATTCATGCAAGGAAACTACCCATAATACAAGAAAAGCCCGTCACGGGCTTCTCAGGGCGTTTTATGGCGGGTCTGCTATGTGGTGCTATCTGACTTTTTGCTGTTCAGCAGTTCCTGCCCTCTGATTTTCCAGTCTGACCACTTCGGATTATCCCGTGACAGGTCATTCAGACTGGCTAATGCACCCAGTAAGGCAGCGGTATCATCAACAGGCTTACCCGTCTTACTGTCCCTAGTGCTTGGATTCTCACCAATAAAAAACGCCCGGCGGCAACCGAGCGTTCTGAACAAATCCAGATGGAGTTCTGAGGTCATTACTGGATCTATCAACAGGAGTCCAAGCGAGCTCTCGAACCCCAGAGTCCCGCTATGTATCCGCTCATGAATTAATTCTTATTACGCCCCGCCCTGCCACTCATCGCAGTACTGTTGTAATTCATTAAGCATTCTGCCGACATGGAAGCCATCACAAACGGCATGATGAACCTGAATCGCCAGCGGCATCAGCACCTTGTCGCCTTGCGTATAATATTTGCCCATGGTGAAAACGGGGGCGAAGAAGTTGTCCATATTGGCCACGTTTAAATCAAAACTGGTGAAACTCACCCAGGGATTGGCTGAGACGAAAAACATATTCTCAATAAACCCTTTAGGGAAATAGGCCAGGTTTTCACCGTAACACGCCACATCTTGCGAATATATGTGTAGAAACTGCCGGAAATCGTCGTGGTATTCACTCCAGAGCGATGAAAACGTTTCAGTTTGCTCATGGAAAACGGTGTAACAAGGGTGAACACTATCCCATATCACCAGCTCACCGTCTTTCATTGCCATACGAAATTCCGGATGAGCATTCATCAGGCGGGCAAGAATGTGAATAAAGGCCGGATAAAACTTGTGCTTATTTTTCTTTACGGTCTTTAAAAAGGCCGTAATATCCAGCTGAACGGTCTGGTTATAGGTACATTGAGCAACTGACTGAAATGCCTCAAAATGTTCTTTACGATGCCATTGGGATATATCAACGGTGGTATATCCAGTGATTTTTTTCTCCATTTTAGCTTCCTTAGCTCCTGAAAATCTCGATAACTCAAAAAATACGCCCGGTAGTGATCTTATTTCATTATGGTGAAAGTTGGAACCTCTTACGTGCCGATCAAGATCAAAGGATCTTCTTGAGATCCCCAGCTGGCAATTCCGAC*

pST42

*CATTAAGCAAATATAACGCCCTGAGAATTTCGACAGGCAAAAGAAAAAGGGGTTAGCATTTAGCTAACCCCTTATCTTATTTGGCGGAAGCGCAGAGATTCGAACTCTGGAACCCTTTCGGGTCGCCGGTTTTCAAGACCGGTGCCTTCAACCGCTCGGCCACACTTCCGGAATGACGCGCACTATAAACATCCCGATGCGGCGTGTAAACCCCTAATTTGTTTGTTTGCCTGAAAAACAGCCAAAAGTGCATTGATAGCGCATGCAAGCTCTAGAGGCATCAAATAAAACGAAAGGCTCAGTCGAAAGACTGGGCCTTTCGTTTTATCTGTTGTTTGTCGGTGAACGCTCTCCTGAGTAGGACAAATCCGCCGCCCTAGACCTAGGGTACGGGTTTTGCTGCCCGCAAACGGGCTGTTCTGGTGTTGCTAGTTTGTTATCAGAATCGCAGATCCGGCTTCAGCCGGTTTGCCGGCTGAAAGCGCTATTTCTTCCAGAATTGCCATGATTTTTTCCCCACGGGAGGCGTCACTGGCTCCCGTGTTGTCGGCAGCTTTGATTCGATAAGCAGCATCGCCTGTTTCAGGCTGTCTATGTGTGACTGTTGAGCTGTAACAAGTTGTCTCAGGTGTTCAATTTCATGTTCTAGTTGCTTTGTTTTACTGGTTTCACCTGTTCTATTAGGTGTTACATGCTGTTCATCTGTTACATTGTCGATCTGTTCATGGTGAACAGCTTTGAATGCACCAAAAACTCGTAAAAGCTCTGATGTATCTATCTTTTTTACACCGTTTTCATCTGTGCATATGGACAGTTTTCCCTTTGATATGTAACGGTGAACAGTTGTTCTACTTTTGTTTGTTAGTCTTGATGCTTCACTGATAGATACAAGAGCCATAAGAACCTCAGATCCTTCCGTATTTAGCCAGTATGTTCTCTAGTGTGGTTCGTTGTTTTTGCGTGAGCCATGAGAACGAACCATTGAGATCATACTTACTTTGCATGTCACTCAAAAATTTTGCCTCAAAACTGGTGAGCTGAATTTTTGCAGTTAAAGCATCGTGTAGTGTTTTTCTTAGTCCGTTATGTAGGTAGGAATCTGATGTAATGGTTGTTGGTATTTTGTCACCATTCATTTTTATCTGGTTGTTCTCAAGTTCGGTTACGAGATCCATTTGTCTATCTAGTTCAACTTGGAAAATCAACGTATCAGTCGGGCGGCCTCGCTTATCAACCACCAATTTCATATTGCTGTAAGTGTTTAAATCTTTACTTATTGGTTTCAAAACCCATTGGTTAAGCCTTTTAAACTCATGGTAGTTATTTTCAAGCATTAACATGAACTTAAATTCATCAAGGCTAATCTCTATATTTGCCTTGTGAGTTTTCTTTTGTGTTAGTTCTTTTAATAACCACTCATAAATCCTCATAGAGTATTTGTTTTCAAAAGACTTAACATGTTCCAGATTATATTTTATGAATTTTTTTAACTGGAAAAGATAAGGCAATATCTCTTCACTAAAAACTAATTCTAATTTTTCGCTTGAGAACTTGGCATAGTTTGTCCACTGGAAAATCTCAAAGCCTTTAACCAAAGGATTCCTGATTTCCACAGTTCTCGTCATCAGCTCTCTGGTTGCTTTAGCTAATACACCATAAGCATTTTCCCTACTGATGTTCATCATCTGAGCGTATTGGTTATAAGTGAACGATACCGTCCGTTCTTTCCTTGTAGGGTTTTCAATCGTGGGGTTGAGTAGTGCCACACAGCATAAAATTAGCTTGGTTTCATGCTCCGTTAAGTCATAGCGACTAATCGCTAGTTCATTTGCTTTGAAAACAACTAATTCAGACATACATCTCAATTGGTCTAGGTGATTTTAATCACTATACCAATTGAGATGGGCTAGTCAATGATAATTACTAGTCCTTTTCCTTTGAGTTGTGGGTATCTGTAAATTCTGCTAGACCTTTGCTGGAAAACTTGTAAATTCTGCTAGACCCTCTGTAAATTCCGCTAGACCTTTGTGTGTTTTTTTTGTTTATATTCAAGTGGTTATAATTTATAGAATAAAGAAAGAATAAAAAAAGATAAAAAGAATAGATCCCAGCCCTGTGTATAACTCACTACTTTAGTCAGTTCCGCAGTATTACAAAAGGATGTCGCAAACGCTGTTTGCTCCTCTACAAAACAGACCTTAAAACCCTAAAGGCTTAAGTAGCGCCGTCGCAAGCTCCGGCAAATCGCTGAATATTCCTTTTGTCTCCGACCATCAGGCACCTGAGTCGCTGTCTTTTTCGTCACATTCAGTTCGCTGCGCTCACGGCTCTGGCAGTGAATGGGGGTAAATGGCACTACAGGCGCCTTTTATGGATTCATGCAAGGAAACTACCCATAATACAAGAAAAGCCCGTCACGGGCTTCTCAGGGCGTTTTATGGCGGGTCTGCTATGTGGTGCTATCTGACTTTTTGCTGTTCAGCAGTTCCTGCCCTCTGATTTTCCAGTCTGACCACTTCGGATTATCCCGTGACAGGTCATTCAGACTGGCTAATGCACCCAGTAAGGCAGCGGTATCATCAACAGGCTTACCCGTCTTACTGTCCCTAGTGCTTGGATTCTCACCAATAAAAAACGCCCGGCGGCAACCGAGCGTTCTGAACAAATCCAGATGGAGTTCTGAGGTCATTACTGGATCTATCAACAGGAGTCCAAGCGAGCTCTCGAACCCCAGAGTCCCGCTATGTATCCGCTCATGAATTAATTCTTATTACGCCCCGCCCTGCCACTCATCGCAGTACTGTTGTAATTCATTAAGCATTCTGCCGACATGGAAGCCATCACAAACGGCATGATGAACCTGAATCGCCAGCGGCATCAGCACCTTGTCGCCTTGCGTATAATATTTGCCCATGGTGAAAACGGGGGCGAAGAAGTTGTCCATATTGGCCACGTTTAAATCAAAACTGGTGAAACTCACCCAGGGATTGGCTGAGACGAAAAACATATTCTCAATAAACCCTTTAGGGAAATAGGCCAGGTTTTCACCGTAACACGCCACATCTTGCGAATATATGTGTAGAAACTGCCGGAAATCGTCGTGGTATTCACTCCAGAGCGATGAAAACGTTTCAGTTTGCTCATGGAAAACGGTGTAACAAGGGTGAACACTATCCCATATCACCAGCTCACCGTCTTTCATTGCCATACGAAATTCCGGATGAGCATTCATCAGGCGGGCAAGAATGTGAATAAAGGCCGGATAAAACTTGTGCTTATTTTTCTTTACGGTCTTTAAAAAGGCCGTAATATCCAGCTGAACGGTCTGGTTATAGGTACATTGAGCAACTGACTGAAATGCCTCAAAATGTTCTTTACGATGCCATTGGGATATATCAACGGTGGTATATCCAGTGATTTTTTTCTCCATTTTAGCTTCCTTAGCTCCTGAAAATCTCGATAACTCAAAAAATACGCCCGGTAGTGATCTTATTTCATTATGGTGAAAGTTGGAACCTCTTACGTGCCGATCAAGATCAAAGGATCTTCTTGAGATCCCCAGCTGGCAATTCCGAC*

pST43

*CGCACAAACCGTAACCAAACGCGCAATTTATTTAAAAAGGGACTAGACAGAGGGGTGGGAAGTCCGTATTATCCACCCCCGCAACGGCGCTAAGCGCCCGTAGCTCAGCTGGATAGAGCGCTGCCCTCCGGAGGCAGAGGTCTCAGGTTCGAATCCTGTCGGGCGCGCCATTTAGTCCCGGCGCTTGAGCTGCGGTGGTAGTAATACCGCGTAACAAGATTTGTAGTGGTGGCTATAGCTCAGTTGGTAGAGCCCTGGATTGTGATTCCAGTTGTCGTGGGTTCGAATCCCATTAGCCACCCCATTATTAGAAGTTGTGACAATGCGAAGGTGGCGGAATTGGTAGACGCGCTAGCTTCAGGTGTTAGTGTCCTTACGGACGTGGGGGTTCAAGTCCCCCCCCTCGCACCACGACTTTAAAGAATTGAACTAAAAATTCAAAAAGCAGTATTTCGGCGAGTAGCGCAGCTTGGTAGCGCAACTGGTTTGGGACCAGTGGGTCGGAGGTTCGAATCCTCTCTCGCCGACCAATTTTGAACCCCGCTTCGGCGGGGTTTTTTGTTTTCTGTGCATTTCGTCACCATGCAAGCTCTAGAGGCATCAAATAAAACGAAAGGCTCAGTCGAAAGACTGGGCCTTTCGTTTTATCTGTTGTTTGTCGGTGAACGCTCTCCTGAGTAGGACAAATCCGCCGCCCTAGACCTAGGGTACGGGTTTTGCTGCCCGCAAACGGGCTGTTCTGGTGTTGCTAGTTTGTTATCAGAATCGCAGATCCGGCTTCAGCCGGTTTGCCGGCTGAAAGCGCTATTTCTTCCAGAATTGCCATGATTTTTTCCCCACGGGAGGCGTCACTGGCTCCCGTGTTGTCGGCAGCTTTGATTCGATAAGCAGCATCGCCTGTTTCAGGCTGTCTATGTGTGACTGTTGAGCTGTAACAAGTTGTCTCAGGTGTTCAATTTCATGTTCTAGTTGCTTTGTTTTACTGGTTTCACCTGTTCTATTAGGTGTTACATGCTGTTCATCTGTTACATTGTCGATCTGTTCATGGTGAACAGCTTTGAATGCACCAAAAACTCGTAAAAGCTCTGATGTATCTATCTTTTTTACACCGTTTTCATCTGTGCATATGGACAGTTTTCCCTTTGATATGTAACGGTGAACAGTTGTTCTACTTTTGTTTGTTAGTCTTGATGCTTCACTGATAGATACAAGAGCCATAAGAACCTCAGATCCTTCCGTATTTAGCCAGTATGTTCTCTAGTGTGGTTCGTTGTTTTTGCGTGAGCCATGAGAACGAACCATTGAGATCATACTTACTTTGCATGTCACTCAAAAATTTTGCCTCAAAACTGGTGAGCTGAATTTTTGCAGTTAAAGCATCGTGTAGTGTTTTTCTTAGTCCGTTATGTAGGTAGGAATCTGATGTAATGGTTGTTGGTATTTTGTCACCATTCATTTTTATCTGGTTGTTCTCAAGTTCGGTTACGAGATCCATTTGTCTATCTAGTTCAACTTGGAAAATCAACGTATCAGTCGGGCGGCCTCGCTTATCAACCACCAATTTCATATTGCTGTAAGTGTTTAAATCTTTACTTATTGGTTTCAAAACCCATTGGTTAAGCCTTTTAAACTCATGGTAGTTATTTTCAAGCATTAACATGAACTTAAATTCATCAAGGCTAATCTCTATATTTGCCTTGTGAGTTTTCTTTTGTGTTAGTTCTTTTAATAACCACTCATAAATCCTCATAGAGTATTTGTTTTCAAAAGACTTAACATGTTCCAGATTATATTTTATGAATTTTTTTAACTGGAAAAGATAAGGCAATATCTCTTCACTAAAAACTAATTCTAATTTTTCGCTTGAGAACTTGGCATAGTTTGTCCACTGGAAAATCTCAAAGCCTTTAACCAAAGGATTCCTGATTTCCACAGTTCTCGTCATCAGCTCTCTGGTTGCTTTAGCTAATACACCATAAGCATTTTCCCTACTGATGTTCATCATCTGAGCGTATTGGTTATAAGTGAACGATACCGTCCGTTCTTTCCTTGTAGGGTTTTCAATCGTGGGGTTGAGTAGTGCCACACAGCATAAAATTAGCTTGGTTTCATGCTCCGTTAAGTCATAGCGACTAATCGCTAGTTCATTTGCTTTGAAAACAACTAATTCAGACATACATCTCAATTGGTCTAGGTGATTTTAATCACTATACCAATTGAGATGGGCTAGTCAATGATAATTACTAGTCCTTTTCCTTTGAGTTGTGGGTATCTGTAAATTCTGCTAGACCTTTGCTGGAAAACTTGTAAATTCTGCTAGACCCTCTGTAAATTCCGCTAGACCTTTGTGTGTTTTTTTTGTTTATATTCAAGTGGTTATAATTTATAGAATAAAGAAAGAATAAAAAAAGATAAAAAGAATAGATCCCAGCCCTGTGTATAACTCACTACTTTAGTCAGTTCCGCAGTATTACAAAAGGATGTCGCAAACGCTGTTTGCTCCTCTACAAAACAGACCTTAAAACCCTAAAGGCTTAAGTAGCGCCGTCGCAAGCTCCGGCAAATCGCTGAATATTCCTTTTGTCTCCGACCATCAGGCACCTGAGTCGCTGTCTTTTTCGTCACATTCAGTTCGCTGCGCTCACGGCTCTGGCAGTGAATGGGGGTAAATGGCACTACAGGCGCCTTTTATGGATTCATGCAAGGAAACTACCCATAATACAAGAAAAGCCCGTCACGGGCTTCTCAGGGCGTTTTATGGCGGGTCTGCTATGTGGTGCTATCTGACTTTTTGCTGTTCAGCAGTTCCTGCCCTCTGATTTTCCAGTCTGACCACTTCGGATTATCCCGTGACAGGTCATTCAGACTGGCTAATGCACCCAGTAAGGCAGCGGTATCATCAACAGGCTTACCCGTCTTACTGTCCCTAGTGCTTGGATTCTCACCAATAAAAAACGCCCGGCGGCAACCGAGCGTTCTGAACAAATCCAGATGGAGTTCTGAGGTCATTACTGGATCTATCAACAGGAGTCCAAGCGAGCTCTCGAACCCCAGAGTCCCGCTATGTATCCGCTCATGAATTAATTCTTATTACGCCCCGCCCTGCCACTCATCGCAGTACTGTTGTAATTCATTAAGCATTCTGCCGACATGGAAGCCATCACAAACGGCATGATGAACCTGAATCGCCAGCGGCATCAGCACCTTGTCGCCTTGCGTATAATATTTGCCCATGGTGAAAACGGGGGCGAAGAAGTTGTCCATATTGGCCACGTTTAAATCAAAACTGGTGAAACTCACCCAGGGATTGGCTGAGACGAAAAACATATTCTCAATAAACCCTTTAGGGAAATAGGCCAGGTTTTCACCGTAACACGCCACATCTTGCGAATATATGTGTAGAAACTGCCGGAAATCGTCGTGGTATTCACTCCAGAGCGATGAAAACGTTTCAGTTTGCTCATGGAAAACGGTGTAACAAGGGTGAACACTATCCCATATCACCAGCTCACCGTCTTTCATTGCCATACGAAATTCCGGATGAGCATTCATCAGGCGGGCAAGAATGTGAATAAAGGCCGGATAAAACTTGTGCTTATTTTTCTTTACGGTCTTTAAAAAGGCCGTAATATCCAGCTGAACGGTCTGGTTATAGGTACATTGAGCAACTGACTGAAATGCCTCAAAATGTTCTTTACGATGCCATTGGGATATATCAACGGTGGTATATCCAGTGATTTTTTTCTCCATTTTAGCTTCCTTAGCTCCTGAAAATCTCGATAACTCAAAAAATACGCCCGGTAGTGATCTTATTTCATTATGGTGAAAGTTGGAACCTCTTACGTGCCGATCAAGATCAAAGGATCTTCTTGAGATCCCCAGCTGGCAATTCCGAC*

pST44

*GGAGCGCATTGTTGAGCACAATGATGTTGAAAAAGTGTGCTAATCTGCCCTCCGTTCGGCTGTTTCTTCATCGTGTCGCATAAAATGTGACCAATAAAACAAATTATGCAATTTTTTAGTTGCATGAACTCGCATGTCTCCATAGAATGCGCGCTACTTGATGCCGACTTAGCTCAGTAGGTAGAGCAACTGACTTGTAATCAGTAGGTCACCAGTTCGATTCCGGTAGTCGGCACCATCAAGTCCGGTGGGGTTCCCGAGCGGCCAAAGGGAGCAGACTGTAAATCTGCCGTCACAGACTTCGAAGGTTCGAATCCTTCCCCCACCACCAATTTCGGCCACGCGATGGCGTAGCCCGAGACGATAAGTTCGCTTACCGGCTCGAATAAAGAGAGCTTCTCTCGATATTCAGTGCAGAATGAAAATCAGGTAGCCGAGTTCCAGGATGCGGGCATCGTATAATGGCTATTACCTCAGCCTTCCAAGCTGATGATGCGGGTTCGATTCCCGCTGCCCGCTCCAAGATGTGCTGATATAGCTCAGTTGGTAGAGCGCACCCTTGGTAAGGGTGAGGTCGGCAGTTCGAATCTGCCTATCAGCACCACTTCTTTTCTCCTCCCTGTTTTTTCCTTCTGTTTATTGCATTCAACAAGTCGGGCATGTTGCCATGCAAGCTCTAGAGGCATCAAATAAAACGAAAGGCTCAGTCGAAAGACTGGGCCTTTCGTTTTATCTGTTGTTTGTCGGTGAACGCTCTCCTGAGTAGGACAAATCCGCCGCCCTAGACCTAGGGTACGGGTTTTGCTGCCCGCAAACGGGCTGTTCTGGTGTTGCTAGTTTGTTATCAGAATCGCAGATCCGGCTTCAGCCGGTTTGCCGGCTGAAAGCGCTATTTCTTCCAGAATTGCCATGATTTTTTCCCCACGGGAGGCGTCACTGGCTCCCGTGTTGTCGGCAGCTTTGATTCGATAAGCAGCATCGCCTGTTTCAGGCTGTCTATGTGTGACTGTTGAGCTGTAACAAGTTGTCTCAGGTGTTCAATTTCATGTTCTAGTTGCTTTGTTTTACTGGTTTCACCTGTTCTATTAGGTGTTACATGCTGTTCATCTGTTACATTGTCGATCTGTTCATGGTGAACAGCTTTGAATGCACCAAAAACTCGTAAAAGCTCTGATGTATCTATCTTTTTTACACCGTTTTCATCTGTGCATATGGACAGTTTTCCCTTTGATATGTAACGGTGAACAGTTGTTCTACTTTTGTTTGTTAGTCTTGATGCTTCACTGATAGATACAAGAGCCATAAGAACCTCAGATCCTTCCGTATTTAGCCAGTATGTTCTCTAGTGTGGTTCGTTGTTTTTGCGTGAGCCATGAGAACGAACCATTGAGATCATACTTACTTTGCATGTCACTCAAAAATTTTGCCTCAAAACTGGTGAGCTGAATTTTTGCAGTTAAAGCATCGTGTAGTGTTTTTCTTAGTCCGTTATGTAGGTAGGAATCTGATGTAATGGTTGTTGGTATTTTGTCACCATTCATTTTTATCTGGTTGTTCTCAAGTTCGGTTACGAGATCCATTTGTCTATCTAGTTCAACTTGGAAAATCAACGTATCAGTCGGGCGGCCTCGCTTATCAACCACCAATTTCATATTGCTGTAAGTGTTTAAATCTTTACTTATTGGTTTCAAAACCCATTGGTTAAGCCTTTTAAACTCATGGTAGTTATTTTCAAGCATTAACATGAACTTAAATTCATCAAGGCTAATCTCTATATTTGCCTTGTGAGTTTTCTTTTGTGTTAGTTCTTTTAATAACCACTCATAAATCCTCATAGAGTATTTGTTTTCAAAAGACTTAACATGTTCCAGATTATATTTTATGAATTTTTTTAACTGGAAAAGATAAGGCAATATCTCTTCACTAAAAACTAATTCTAATTTTTCGCTTGAGAACTTGGCATAGTTTGTCCACTGGAAAATCTCAAAGCCTTTAACCAAAGGATTCCTGATTTCCACAGTTCTCGTCATCAGCTCTCTGGTTGCTTTAGCTAATACACCATAAGCATTTTCCCTACTGATGTTCATCATCTGAGCGTATTGGTTATAAGTGAACGATACCGTCCGTTCTTTCCTTGTAGGGTTTTCAATCGTGGGGTTGAGTAGTGCCACACAGCATAAAATTAGCTTGGTTTCATGCTCCGTTAAGTCATAGCGACTAATCGCTAGTTCATTTGCTTTGAAAACAACTAATTCAGACATACATCTCAATTGGTCTAGGTGATTTTAATCACTATACCAATTGAGATGGGCTAGTCAATGATAATTACTAGTCCTTTTCCTTTGAGTTGTGGGTATCTGTAAATTCTGCTAGACCTTTGCTGGAAAACTTGTAAATTCTGCTAGACCCTCTGTAAATTCCGCTAGACCTTTGTGTGTTTTTTTTGTTTATATTCAAGTGGTTATAATTTATAGAATAAAGAAAGAATAAAAAAAGATAAAAAGAATAGATCCCAGCCCTGTGTATAACTCACTACTTTAGTCAGTTCCGCAGTATTACAAAAGGATGTCGCAAACGCTGTTTGCTCCTCTACAAAACAGACCTTAAAACCCTAAAGGCTTAAGTAGCGCCGTCGCAAGCTCCGGCAAATCGCTGAATATTCCTTTTGTCTCCGACCATCAGGCACCTGAGTCGCTGTCTTTTTCGTCACATTCAGTTCGCTGCGCTCACGGCTCTGGCAGTGAATGGGGGTAAATGGCACTACAGGCGCCTTTTATGGATTCATGCAAGGAAACTACCCATAATACAAGAAAAGCCCGTCACGGGCTTCTCAGGGCGTTTTATGGCGGGTCTGCTATGTGGTGCTATCTGACTTTTTGCTGTTCAGCAGTTCCTGCCCTCTGATTTTCCAGTCTGACCACTTCGGATTATCCCGTGACAGGTCATTCAGACTGGCTAATGCACCCAGTAAGGCAGCGGTATCATCAACAGGCTTACCCGTCTTACTGTCCCTAGTGCTTGGATTCTCACCAATAAAAAACGCCCGGCGGCAACCGAGCGTTCTGAACAAATCCAGATGGAGTTCTGAGGTCATTACTGGATCTATCAACAGGAGTCCAAGCGAGCTCTCGAACCCCAGAGTCCCGCTATGTATCCGCTCATGAATTAATTCTTATTACGCCCCGCCCTGCCACTCATCGCAGTACTGTTGTAATTCATTAAGCATTCTGCCGACATGGAAGCCATCACAAACGGCATGATGAACCTGAATCGCCAGCGGCATCAGCACCTTGTCGCCTTGCGTATAATATTTGCCCATGGTGAAAACGGGGGCGAAGAAGTTGTCCATATTGGCCACGTTTAAATCAAAACTGGTGAAACTCACCCAGGGATTGGCTGAGACGAAAAACATATTCTCAATAAACCCTTTAGGGAAATAGGCCAGGTTTTCACCGTAACACGCCACATCTTGCGAATATATGTGTAGAAACTGCCGGAAATCGTCGTGGTATTCACTCCAGAGCGATGAAAACGTTTCAGTTTGCTCATGGAAAACGGTGTAACAAGGGTGAACACTATCCCATATCACCAGCTCACCGTCTTTCATTGCCATACGAAATTCCGGATGAGCATTCATCAGGCGGGCAAGAATGTGAATAAAGGCCGGATAAAACTTGTGCTTATTTTTCTTTACGGTCTTTAAAAAGGCCGTAATATCCAGCTGAACGGTCTGGTTATAGGTACATTGAGCAACTGACTGAAATGCCTCAAAATGTTCTTTACGATGCCATTGGGATATATCAACGGTGGTATATCCAGTGATTTTTTTCTCCATTTTAGCTTCCTTAGCTCCTGAAAATCTCGATAACTCAAAAAATACGCCCGGTAGTGATCTTATTTCATTATGGTGAAAGTTGGAACCTCTTACGTGCCGATCAAGATCAAAGGATCTTCTTGAGATCCCCAGCTGGCAATTCCGAC*

pST45

*GTGTAACGACAAGTTGCAGGCACAAAAAAACCACCCGAAGGTGGTTTCACGACACTGCTTATTGCTTTGATTTTATTCTTATCTTTCCCATGGTACCCGGAGCGGGACTTGAACCCGCACAGCGCGAACGCCGAGGGATTTTAAATCCCTTGTGTCTACCGATTCCACCATCCGGGCTCGGGAAGAAAGTGGAGGCGCGTTCCGGAGTCGAACCGGACTAGACGGATTTGCAATCCGCTACATAACCGCTTTGTTAACGCGCCAAATTCTTCAGGCCTTTCAGCCAGACATCCGCTTGACGCCGATGTCTTTTAAACTGGAGCGGGAAACGAGACTCGAACTCGCGACCCCGACCTTGGCAAGGTCGTGCTCTACCAACTGAGCTATTCCCGCATTCATCAAGCAATCAGTTAATCACTTGATTTTATTATCGTCTGGCAATCAGTGCCGCCGTTCGATGCGTTGCATTCTACTTACCTGGCGCGATGAGTCAACCATGCAAGCTCTAGAGGCATCAAATAAAACGAAAGGCTCAGTCGAAAGACTGGGCCTTTCGTTTTATCTGTTGTTTGTCGGTGAACGCTCTCCTGAGTAGGACAAATCCGCCGCCCTAGACCTAGGGTACGGGTTTTGCTGCCCGCAAACGGGCTGTTCTGGTGTTGCTAGTTTGTTATCAGAATCGCAGATCCGGCTTCAGCCGGTTTGCCGGCTGAAAGCGCTATTTCTTCCAGAATTGCCATGATTTTTTCCCCACGGGAGGCGTCACTGGCTCCCGTGTTGTCGGCAGCTTTGATTCGATAAGCAGCATCGCCTGTTTCAGGCTGTCTATGTGTGACTGTTGAGCTGTAACAAGTTGTCTCAGGTGTTCAATTTCATGTTCTAGTTGCTTTGTTTTACTGGTTTCACCTGTTCTATTAGGTGTTACATGCTGTTCATCTGTTACATTGTCGATCTGTTCATGGTGAACAGCTTTGAATGCACCAAAAACTCGTAAAAGCTCTGATGTATCTATCTTTTTTACACCGTTTTCATCTGTGCATATGGACAGTTTTCCCTTTGATATGTAACGGTGAACAGTTGTTCTACTTTTGTTTGTTAGTCTTGATGCTTCACTGATAGATACAAGAGCCATAAGAACCTCAGATCCTTCCGTATTTAGCCAGTATGTTCTCTAGTGTGGTTCGTTGTTTTTGCGTGAGCCATGAGAACGAACCATTGAGATCATACTTACTTTGCATGTCACTCAAAAATTTTGCCTCAAAACTGGTGAGCTGAATTTTTGCAGTTAAAGCATCGTGTAGTGTTTTTCTTAGTCCGTTATGTAGGTAGGAATCTGATGTAATGGTTGTTGGTATTTTGTCACCATTCATTTTTATCTGGTTGTTCTCAAGTTCGGTTACGAGATCCATTTGTCTATCTAGTTCAACTTGGAAAATCAACGTATCAGTCGGGCGGCCTCGCTTATCAACCACCAATTTCATATTGCTGTAAGTGTTTAAATCTTTACTTATTGGTTTCAAAACCCATTGGTTAAGCCTTTTAAACTCATGGTAGTTATTTTCAAGCATTAACATGAACTTAAATTCATCAAGGCTAATCTCTATATTTGCCTTGTGAGTTTTCTTTTGTGTTAGTTCTTTTAATAACCACTCATAAATCCTCATAGAGTATTTGTTTTCAAAAGACTTAACATGTTCCAGATTATATTTTATGAATTTTTTTAACTGGAAAAGATAAGGCAATATCTCTTCACTAAAAACTAATTCTAATTTTTCGCTTGAGAACTTGGCATAGTTTGTCCACTGGAAAATCTCAAAGCCTTTAACCAAAGGATTCCTGATTTCCACAGTTCTCGTCATCAGCTCTCTGGTTGCTTTAGCTAATACACCATAAGCATTTTCCCTACTGATGTTCATCATCTGAGCGTATTGGTTATAAGTGAACGATACCGTCCGTTCTTTCCTTGTAGGGTTTTCAATCGTGGGGTTGAGTAGTGCCACACAGCATAAAATTAGCTTGGTTTCATGCTCCGTTAAGTCATAGCGACTAATCGCTAGTTCATTTGCTTTGAAAACAACTAATTCAGACATACATCTCAATTGGTCTAGGTGATTTTAATCACTATACCAATTGAGATGGGCTAGTCAATGATAATTACTAGTCCTTTTCCTTTGAGTTGTGGGTATCTGTAAATTCTGCTAGACCTTTGCTGGAAAACTTGTAAATTCTGCTAGACCCTCTGTAAATTCCGCTAGACCTTTGTGTGTTTTTTTTGTTTATATTCAAGTGGTTATAATTTATAGAATAAAGAAAGAATAAAAAAAGATAAAAAGAATAGATCCCAGCCCTGTGTATAACTCACTACTTTAGTCAGTTCCGCAGTATTACAAAAGGATGTCGCAAACGCTGTTTGCTCCTCTACAAAACAGACCTTAAAACCCTAAAGGCTTAAGTAGCGCCGTCGCAAGCTCCGGCAAATCGCTGAATATTCCTTTTGTCTCCGACCATCAGGCACCTGAGTCGCTGTCTTTTTCGTCACATTCAGTTCGCTGCGCTCACGGCTCTGGCAGTGAATGGGGGTAAATGGCACTACAGGCGCCTTTTATGGATTCATGCAAGGAAACTACCCATAATACAAGAAAAGCCCGTCACGGGCTTCTCAGGGCGTTTTATGGCGGGTCTGCTATGTGGTGCTATCTGACTTTTTGCTGTTCAGCAGTTCCTGCCCTCTGATTTTCCAGTCTGACCACTTCGGATTATCCCGTGACAGGTCATTCAGACTGGCTAATGCACCCAGTAAGGCAGCGGTATCATCAACAGGCTTACCCGTCTTACTGTCCCTAGTGCTTGGATTCTCACCAATAAAAAACGCCCGGCGGCAACCGAGCGTTCTGAACAAATCCAGATGGAGTTCTGAGGTCATTACTGGATCTATCAACAGGAGTCCAAGCGAGCTCTCGAACCCCAGAGTCCCGCTATGTATCCGCTCATGAATTAATTCTTATTACGCCCCGCCCTGCCACTCATCGCAGTACTGTTGTAATTCATTAAGCATTCTGCCGACATGGAAGCCATCACAAACGGCATGATGAACCTGAATCGCCAGCGGCATCAGCACCTTGTCGCCTTGCGTATAATATTTGCCCATGGTGAAAACGGGGGCGAAGAAGTTGTCCATATTGGCCACGTTTAAATCAAAACTGGTGAAACTCACCCAGGGATTGGCTGAGACGAAAAACATATTCTCAATAAACCCTTTAGGGAAATAGGCCAGGTTTTCACCGTAACACGCCACATCTTGCGAATATATGTGTAGAAACTGCCGGAAATCGTCGTGGTATTCACTCCAGAGCGATGAAAACGTTTCAGTTTGCTCATGGAAAACGGTGTAACAAGGGTGAACACTATCCCATATCACCAGCTCACCGTCTTTCATTGCCATACGAAATTCCGGATGAGCATTCATCAGGCGGGCAAGAATGTGAATAAAGGCCGGATAAAACTTGTGCTTATTTTTCTTTACGGTCTTTAAAAAGGCCGTAATATCCAGCTGAACGGTCTGGTTATAGGTACATTGAGCAACTGACTGAAATGCCTCAAAATGTTCTTTACGATGCCATTGGGATATATCAACGGTGGTATATCCAGTGATTTTTTTCTCCATTTTAGCTTCCTTAGCTCCTGAAAATCTCGATAACTCAAAAAATACGCCCGGTAGTGATCTTATTTCATTATGGTGAAAGTTGGAACCTCTTACGTGCCGATCAAGATCAAAGGATCTTCTTGAGATCCCCAGCTGGCAATTCCGAC*

pST46

*CGCGCGGTAATGGCTGGATTGCGACACGGAGTTACTTTATAATCCGCTACCATGGCCCCTTAGCTCAGTGGTTAGAGCAGGCGACTCATAATCGCTTGGTCGCTGGTTCAAGTCCAGCAGGGGCCACCAGATATAGCAAAGGCTGACGAGAAATCGTCAGCCTTTTTCTTTTTATATATCACATGCAAGCTCTAGAGGCATCAAATAAAACGAAAGGCTCAGTCGAAAGACTGGGCCTTTCGTTTTATCTGTTGTTTGTCGGTGAACGCTCTCCTGAGTAGGACAAATCCGCCGCCCTAGACCTAGGGTACGGGTTTTGCTGCCCGCAAACGGGCTGTTCTGGTGTTGCTAGTTTGTTATCAGAATCGCAGATCCGGCTTCAGCCGGTTTGCCGGCTGAAAGCGCTATTTCTTCCAGAATTGCCATGATTTTTTCCCCACGGGAGGCGTCACTGGCTCCCGTGTTGTCGGCAGCTTTGATTCGATAAGCAGCATCGCCTGTTTCAGGCTGTCTATGTGTGACTGTTGAGCTGTAACAAGTTGTCTCAGGTGTTCAATTTCATGTTCTAGTTGCTTTGTTTTACTGGTTTCACCTGTTCTATTAGGTGTTACATGCTGTTCATCTGTTACATTGTCGATCTGTTCATGGTGAACAGCTTTGAATGCACCAAAAACTCGTAAAAGCTCTGATGTATCTATCTTTTTTACACCGTTTTCATCTGTGCATATGGACAGTTTTCCCTTTGATATGTAACGGTGAACAGTTGTTCTACTTTTGTTTGTTAGTCTTGATGCTTCACTGATAGATACAAGAGCCATAAGAACCTCAGATCCTTCCGTATTTAGCCAGTATGTTCTCTAGTGTGGTTCGTTGTTTTTGCGTGAGCCATGAGAACGAACCATTGAGATCATACTTACTTTGCATGTCACTCAAAAATTTTGCCTCAAAACTGGTGAGCTGAATTTTTGCAGTTAAAGCATCGTGTAGTGTTTTTCTTAGTCCGTTATGTAGGTAGGAATCTGATGTAATGGTTGTTGGTATTTTGTCACCATTCATTTTTATCTGGTTGTTCTCAAGTTCGGTTACGAGATCCATTTGTCTATCTAGTTCAACTTGGAAAATCAACGTATCAGTCGGGCGGCCTCGCTTATCAACCACCAATTTCATATTGCTGTAAGTGTTTAAATCTTTACTTATTGGTTTCAAAACCCATTGGTTAAGCCTTTTAAACTCATGGTAGTTATTTTCAAGCATTAACATGAACTTAAATTCATCAAGGCTAATCTCTATATTTGCCTTGTGAGTTTTCTTTTGTGTTAGTTCTTTTAATAACCACTCATAAATCCTCATAGAGTATTTGTTTTCAAAAGACTTAACATGTTCCAGATTATATTTTATGAATTTTTTTAACTGGAAAAGATAAGGCAATATCTCTTCACTAAAAACTAATTCTAATTTTTCGCTTGAGAACTTGGCATAGTTTGTCCACTGGAAAATCTCAAAGCCTTTAACCAAAGGATTCCTGATTTCCACAGTTCTCGTCATCAGCTCTCTGGTTGCTTTAGCTAATACACCATAAGCATTTTCCCTACTGATGTTCATCATCTGAGCGTATTGGTTATAAGTGAACGATACCGTCCGTTCTTTCCTTGTAGGGTTTTCAATCGTGGGGTTGAGTAGTGCCACACAGCATAAAATTAGCTTGGTTTCATGCTCCGTTAAGTCATAGCGACTAATCGCTAGTTCATTTGCTTTGAAAACAACTAATTCAGACATACATCTCAATTGGTCTAGGTGATTTTAATCACTATACCAATTGAGATGGGCTAGTCAATGATAATTACTAGTCCTTTTCCTTTGAGTTGTGGGTATCTGTAAATTCTGCTAGACCTTTGCTGGAAAACTTGTAAATTCTGCTAGACCCTCTGTAAATTCCGCTAGACCTTTGTGTGTTTTTTTTGTTTATATTCAAGTGGTTATAATTTATAGAATAAAGAAAGAATAAAAAAAGATAAAAAGAATAGATCCCAGCCCTGTGTATAACTCACTACTTTAGTCAGTTCCGCAGTATTACAAAAGGATGTCGCAAACGCTGTTTGCTCCTCTACAAAACAGACCTTAAAACCCTAAAGGCTTAAGTAGCGCCGTCGCAAGCTCCGGCAAATCGCTGAATATTCCTTTTGTCTCCGACCATCAGGCACCTGAGTCGCTGTCTTTTTCGTCACATTCAGTTCGCTGCGCTCACGGCTCTGGCAGTGAATGGGGGTAAATGGCACTACAGGCGCCTTTTATGGATTCATGCAAGGAAACTACCCATAATACAAGAAAAGCCCGTCACGGGCTTCTCAGGGCGTTTTATGGCGGGTCTGCTATGTGGTGCTATCTGACTTTTTGCTGTTCAGCAGTTCCTGCCCTCTGATTTTCCAGTCTGACCACTTCGGATTATCCCGTGACAGGTCATTCAGACTGGCTAATGCACCCAGTAAGGCAGCGGTATCATCAACAGGCTTACCCGTCTTACTGTCCCTAGTGCTTGGATTCTCACCAATAAAAAACGCCCGGCGGCAACCGAGCGTTCTGAACAAATCCAGATGGAGTTCTGAGGTCATTACTGGATCTATCAACAGGAGTCCAAGCGAGCTCTCGAACCCCAGAGTCCCGCTATGTATCCGCTCATGAATTAATTCTTATTACGCCCCGCCCTGCCACTCATCGCAGTACTGTTGTAATTCATTAAGCATTCTGCCGACATGGAAGCCATCACAAACGGCATGATGAACCTGAATCGCCAGCGGCATCAGCACCTTGTCGCCTTGCGTATAATATTTGCCCATGGTGAAAACGGGGGCGAAGAAGTTGTCCATATTGGCCACGTTTAAATCAAAACTGGTGAAACTCACCCAGGGATTGGCTGAGACGAAAAACATATTCTCAATAAACCCTTTAGGGAAATAGGCCAGGTTTTCACCGTAACACGCCACATCTTGCGAATATATGTGTAGAAACTGCCGGAAATCGTCGTGGTATTCACTCCAGAGCGATGAAAACGTTTCAGTTTGCTCATGGAAAACGGTGTAACAAGGGTGAACACTATCCCATATCACCAGCTCACCGTCTTTCATTGCCATACGAAATTCCGGATGAGCATTCATCAGGCGGGCAAGAATGTGAATAAAGGCCGGATAAAACTTGTGCTTATTTTTCTTTACGGTCTTTAAAAAGGCCGTAATATCCAGCTGAACGGTCTGGTTATAGGTACATTGAGCAACTGACTGAAATGCCTCAAAATGTTCTTTACGATGCCATTGGGATATATCAACGGTGGTATATCCAGTGATTTTTTTCTCCATTTTAGCTTCCTTAGCTCCTGAAAATCTCGATAACTCAAAAAATACGCCCGGTAGTGATCTTATTTCATTATGGTGAAAGTTGGAACCTCTTACGTGCCGATCAAGATCAAAGGATCTTCTTGAGATCCCCAGCTGGCAATTCCGAC*

pST51

CATTAAGCAAATATAACGCCCTGAGAATTTCGACAGGCAAAAGAAAAAGGGGTTAGCATTTAGCTAACCCCTTATCTTATTTGGCGGAAGCGCAGAGATTCGAACTCTGGAACCCTTTCGGGTCGCCGGTTTTCAAGACCGGTGCCTTCAACCGCTCGGCCACACTTCCGGAATGACGCGCACTATAAACATCCCGATGCGGCGTGTAAACCCCTAATTTGTTTGTTTGCCTGAAAAACAGCCAAAAGTGCATTGATAGCGCATGCAAGCTCTAGAGGCATCAAATAAAACGAAAGGCTCAGTCGAAAGACTGGGCCTTTCGTTTTATCTGTTGTTTGTCGGTGAACGCTCTCCTGAGTAGGACAAATCCGCCGCCCTAGACCTAGGGTACGGGTTTTGCTGCCCGCAAACGGGCTGTTCTGGTGTTGCTAGTTTGTTATCAGAATCGCAGATCCGGCTTCAGCCGGTTTGCCGGCTGAAAGCGCTATTTCTTCCAGAATTGCCATGATTTTTTCCCCACGGGAGGCGTCACTGGCTCCCGTGTTGTCGGCAGCTTTGATTCGATAAGCAGCATCGCCTGTTTCAGGCTGTCTATGTGTGACTGTTGAGCTGTAACAAGTTGTCTCAGGTGTTCAATTTCATGTTCTAGTTGCTTTGTTTTACTGGTTTCACCTGTTCTATTAGGTGTTACATGCTGTTCATCTGTTACATTGTCGATCTGTTCATGGTGAACAGCTTTGAATGCACCAAAAACTCGTAAAAGCTCTGATGTATCTATCTTTTTTACACCGTTTTCATCTGTGCATATGGACAGTTTTCCCTTTGATATGTAACGGTGAACAGTTGTTCTACTTTTGTTTGTTAGTCTTGATGCTTCACTGATAGATACAAGAGCCATAAGAACCTCAGATCCTTCCGTATTTAGCCAGTATGTTCTCTAGTGTGGTTCGTTGTTTTTGCGTGAGCCATGAGAACGAACCATTGAGATCATACTTACTTTGCATGTCACTCAAAAATTTTGCCTCAAAACTGGTGAGCTGAATTTTTGCAGTTAAAGCATCGTGTAGTGTTTTTCTTAGTCCGTTATGTAGGTAGGAATCTGATGTAATGGTTGTTGGTATTTTGTCACCATTCATTTTTATCTGGTTGTTCTCAAGTTCGGTTACGAGATCCATTTGTCTATCTAGTTCAACTTGGAAAATCAACGTATCAGTCGGGCGGCCTCGCTTATCAACCACCAATTTCATATTGCTGTAAGTGTTTAAATCTTTACTTATTGGTTTCAAAACCCATTGGTTAAGCCTTTTAAACTCATGGTAGTTATTTTCAAGCATTAACATGAACTTAAATTCATCAAGGCTAATCTCTATATTTGCCTTGTGAGTTTTCTTTTGTGTTAGTTCTTTTAATAACCACTCATAAATCCTCATAGAGTATTTGTTTTCAAAAGACTTAACATGTTCCAGATTATATTTTATGAATTTTTTTAACTGGAAAAGATAAGGCAATATCTCTTCACTAAAAACTAATTCTAATTTTTCGCTTGAGAACTTGGCATAGTTTGTCCACTGGAAAATCTCAAAGCCTTTAACCAAAGGATTCCTGATTTCCACAGTTCTCGTCATCAGCTCTCTGGTTGCTTTAGCTAATACACCATAAGCATTTTCCCTACTGATGTTCATCATCTGAGCGTATTGGTTATAAGTGAACGATACCGTCCGTTCTTTCCTTGTAGGGTTTTCAATCGTGGGGTTGAGTAGTGCCACACAGCATAAAATTAGCTTGGTTTCATGCTCCGTTAAGTCATAGCGACTAATCGCTAGTTCATTTGCTTTGAAAACAACTAATTCAGACATACATCTCAATTGGTCTAGGTGATTTTAATCACTATACCAATTGAGATGGGCTAGTCAATGATAATTACTAGTCCTTTTCCTTTGAGTTGTGGGTATCTGTAAATTCTGCTAGACCTTTGCTGGAAAACTTGTAAATTCTGCTAGACCCTCTGTAAATTCCGCTAGACCTTTGTGTGTTTTTTTTGTTTATATTCAAGTGGTTATAATTTATAGAATAAAGAAAGAATAAAAAAAGATAAAAAGAATAGATCCCAGCCCTGTGTATAACTCACTACTTTAGTCAGTTCCGCAGTATTACAAAAGGATGTCGCAAACGCTGTTTGCTCCTCTACAAAACAGACCTTAAAACCCTAAAGGCTTAAGTAGCGCCGTCGCAAGCTCCGGCAAATCGCTGAATATTCCTTTTGTCTCCGACCATCAGGCACCTGAGTCGCTGTCTTTTTCGTCACATTCAGTTCGCTGCGCTCACGGCTCTGGCAGTGAATGGGGGTAAATGGCACTACAGGCGCCTTTTATGGATTCATGCAAGGAAACTACCCATAATACAAGAAAAGCCCGTCACGGGCTTCTCAGGGCGTTTTATGGCGGGTCTGCTATGTGGTGCTATCTGACTTTTTGCTGTTCAGCAGTTCCTGCCCTCTGATTTTCCAGTCTGACCACTTCGGATTATCCCGTGACAGGTCATTCAGACTGGCTAATGCACCCAGTAAGGCAGCGGTATCATCAACAGGCTTACCCGTCTTACTGTCCCTAGTGCTTGGATTCTCACCAATAAAAAACGCCCGGCGGCAACCGAGCGTTCTGAACAAATCCAGATGGAGTTCTGAGGTCATTACTGGATCTATCAACAGGAGTCCAAGCGAGCTCTCGAACCCCAGAGTCCCGCTATGTATCCGCTCATGAATTAATTCTTATTACGCCCCGCCCTGCCACTCATCGCAGTACTGTTGTAATTCATTAAGCATTCTGCCGACATGGAAGCCATCACAAACGGCATGATGAACCTGAATCGCCAGCGGCATCAGCACCTTGTCGCCTTGCGTATAATATTTGCCCATGGTGAAAACGGGGGCGAAGAAGTTGTCCATATTGGCCACGTTTAAATCAAAACTGGTGAAACTCACCCAGGGATTGGCTGAGACGAAAAACATATTCTCAATAAACCCTTTAGGGAAATAGGCCAGGTTTTCACCGTAACACGCCACATCTTGCGAATATATGTGTAGAAACTGCCGGAAATCGTCGTGGTATTCACTCCAGAGCGATGAAAACGTTTCAGTTTGCTCATGGAAAACGGTGTAACAAGGGTGAACACTATCCCATATCACCAGCTCACCGTCTTTCATTGCCATACGAAATTCCGGATGAGCATTCATCAGGCGGGCAAGAATGTGAATAAAGGCCGGATAAAACTTGTGCTTATTTTTCTTTACGGTCTTTAAAAAGGCCGTAATATCCAGCTGAACGGTCTGGTTATAGGTACATTGAGCAACTGACTGAAATGCCTCAAAATGTTCTTTACGATGCCATTGGGATATATCAACGGTGGTATATCCAGTGATTTTTTTCTCCATTTTAGCTTCCTTAGCTCCTGAAAATCTCGATAACTCAAAAAATACGCCCGGTAGTGATCTTATTTCATTATGGTGAAAGTTGGAACCTCTTACGTGCCGATCAAGATCAAAGGATCTTCTTGAGATCCCCAGCTGGCAATTCCGAC

pST72

CTACACCGAGTGTGGATCTGCCCCATCAAAAAAATATTCTCAACATAAAAAACTTTGTGTAATACTTGTAACGCTGCGCCCTTAGCTCAGTTGGATAGAGCAACGACCTTCTAAGTCGTGGGCCGCAGGTTCGAATCCTGCAGGGCGCGCCAATCCTTAGCGAAAGCTAAGGATTTTTTTTCCTGACTGCTCAAGAACTTACATGCAAGCTCTAGAGGCATCAAATAAAACGAAAGGCTCAGTCGAAAGACTGGGCCTTTCGTTTTATCTGTTGTTTGTCGGTGAACGCTCTCCTGAGTAGGACAAATCCGCCGCCCTAGACCTAGGGTACGGGTTTTGCTGCCCGCAAACGGGCTGTTCTGGTGTTGCTAGTTTGTTATCAGAATCGCAGATCCGGCTTCAGCCGGTTTGCCGGCTGAAAGCGCTATTTCTTCCAGAATTGCCATGATTTTTTCCCCACGGGAGGCGTCACTGGCTCCCGTGTTGTCGGCAGCTTTGATTCGATAAGCAGCATCGCCTGTTTCAGGCTGTCTATGTGTGACTGTTGAGCTGTAACAAGTTGTCTCAGGTGTTCAATTTCATGTTCTAGTTGCTTTGTTTTACTGGTTTCACCTGTTCTATTAGGTGTTACATGCTGTTCATCTGTTACATTGTCGATCTGTTCATGGTGAACAGCTTTGAATGCACCAAAAACTCGTAAAAGCTCTGATGTATCTATCTTTTTTACACCGTTTTCATCTGTGCATATGGACAGTTTTCCCTTTGATATGTAACGGTGAACAGTTGTTCTACTTTTGTTTGTTAGTCTTGATGCTTCACTGATAGATACAAGAGCCATAAGAACCTCAGATCCTTCCGTATTTAGCCAGTATGTTCTCTAGTGTGGTTCGTTGTTTTTGCGTGAGCCATGAGAACGAACCATTGAGATCATACTTACTTTGCATGTCACTCAAAAATTTTGCCTCAAAACTGGTGAGCTGAATTTTTGCAGTTAAAGCATCGTGTAGTGTTTTTCTTAGTCCGTTATGTAGGTAGGAATCTGATGTAATGGTTGTTGGTATTTTGTCACCATTCATTTTTATCTGGTTGTTCTCAAGTTCGGTTACGAGATCCATTTGTCTATCTAGTTCAACTTGGAAAATCAACGTATCAGTCGGGCGGCCTCGCTTATCAACCACCAATTTCATATTGCTGTAAGTGTTTAAATCTTTACTTATTGGTTTCAAAACCCATTGGTTAAGCCTTTTAAACTCATGGTAGTTATTTTCAAGCATTAACATGAACTTAAATTCATCAAGGCTAATCTCTATATTTGCCTTGTGAGTTTTCTTTTGTGTTAGTTCTTTTAATAACCACTCATAAATCCTCATAGAGTATTTGTTTTCAAAAGACTTAACATGTTCCAGATTATATTTTATGAATTTTTTTAACTGGAAAAGATAAGGCAATATCTCTTCACTAAAAACTAATTCTAATTTTTCGCTTGAGAACTTGGCATAGTTTGTCCACTGGAAAATCTCAAAGCCTTTAACCAAAGGATTCCTGATTTCCACAGTTCTCGTCATCAGCTCTCTGGTTGCTTTAGCTAATACACCATAAGCATTTTCCCTACTGATGTTCATCATCTGAGCGTATTGGTTATAAGTGAACGATACCGTCCGTTCTTTCCTTGTAGGGTTTTCAATCGTGGGGTTGAGTAGTGCCACACAGCATAAAATTAGCTTGGTTTCATGCTCCGTTAAGTCATAGCGACTAATCGCTAGTTCATTTGCTTTGAAAACAACTAATTCAGACATACATCTCAATTGGTCTAGGTGATTTTAATCACTATACCAATTGAGATGGGCTAGTCAATGATAATTACTAGTCCTTTTCCTTTGAGTTGTGGGTATCTGTAAATTCTGCTAGACCTTTGCTGGAAAACTTGTAAATTCTGCTAGACCCTCTGTAAATTCCGCTAGACCTTTGTGTGTTTTTTTTGTTTATATTCAAGTGGTTATAATTTATAGAATAAAGAAAGAATAAAAAAAGATAAAAAGAATAGATCCCAGCCCTGTGTATAACTCACTACTTTAGTCAGTTCCGCAGTATTACAAAAGGATGTCGCAAACGCTGTTTGCTCCTCTACAAAACAGACCTTAAAACCCTAAAGGCTTAAGTAGCGCCGTCGCAAGCTCCGGCAAATCGCTGAATATTCCTTTTGTCTCCGACCATCAGGCACCTGAGTCGCTGTCTTTTTCGTCACATTCAGTTCGCTGCGCTCACGGCTCTGGCAGTGAATGGGGGTAAATGGCACTACAGGCGCCTTTTATGGATTCATGCAAGGAAACTACCCATAATACAAGAAAAGCCCGTCACGGGCTTCTCAGGGCGTTTTATGGCGGGTCTGCTATGTGGTGCTATCTGACTTTTTGCTGTTCAGCAGTTCCTGCCCTCTGATTTTCCAGTCTGACCACTTCGGATTATCCCGTGACAGGTCATTCAGACTGGCTAATGCACCCAGTAAGGCAGCGGTATCATCAACAGGCTTACCCGTCTTACTGTCCCTAGTGCTTGGATTCTCACCAATAAAAAACGCCCGGCGGCAACCGAGCGTTCTGAACAAATCCAGATGGAGTTCTGAGGTCATTACTGGATCTATCAACAGGAGTCCAAGCGAGCTCTCGAACCCCAGAGTCCCGCTATGTATCCGCTCATGAATTAATTCTTATTACGCCCCGCCCTGCCACTCATCGCAGTACTGTTGTAATTCATTAAGCATTCTGCCGACATGGAAGCCATCACAAACGGCATGATGAACCTGAATCGCCAGCGGCATCAGCACCTTGTCGCCTTGCGTATAATATTTGCCCATGGTGAAAACGGGGGCGAAGAAGTTGTCCATATTGGCCACGTTTAAATCAAAACTGGTGAAACTCACCCAGGGATTGGCTGAGACGAAAAACATATTCTCAATAAACCCTTTAGGGAAATAGGCCAGGTTTTCACCGTAACACGCCACATCTTGCGAATATATGTGTAGAAACTGCCGGAAATCGTCGTGGTATTCACTCCAGAGCGATGAAAACGTTTCAGTTTGCTCATGGAAAACGGTGTAACAAGGGTGAACACTATCCCATATCACCAGCTCACCGTCTTTCATTGCCATACGAAATTCCGGATGAGCATTCATCAGGCGGGCAAGAATGTGAATAAAGGCCGGATAAAACTTGTGCTTATTTTTCTTTACGGTCTTTAAAAAGGCCGTAATATCCAGCTGAACGGTCTGGTTATAGGTACATTGAGCAACTGACTGAAATGCCTCAAAATGTTCTTTACGATGCCATTGGGATATATCAACGGTGGTATATCCAGTGATTTTTTTCTCCATTTTAGCTTCCTTAGCTCCTGAAAATCTCGATAACTCAAAAAATACGCCCGGTAGTGATCTTATTTCATTATGGTGAAAGTTGGAACCTCTTACGTGCCGATCAAGATCAAAGGATCTTCTTGAGATCCCCAGCTGGCAATTCCGAC

pST73

CTGTATTAGTCGTCCGTAGTGCCCATCAAAAAAATATTCTCAACATAAAAAACTTTGTGTAATACTTGTAACGCTGGAGCGGTAGTTCAGTCGGTTAGAATACCTGCCTGTCACGCAGGGGGTCGCGGGTTCGAGTCCCGTCCGTTCCGCCACCCTAATTAGGGGCGTAGTTCAATTGGTAGAGCACCGGTCTCCAAAACCGGGTGTTGGGAGTTCGAGTCTCTCCGCCCCTGCCATCTCTGTAGTGATTAAGAGCGTGATAAGCAATTTTCGTGTCCCCTTCGTCTAGAGGCCCAGGACACCGCCCTTTCACGGCGGTAACAGGGGTTCGAATCCCCTAGGGGACGCCAATCCTTAGCGAAAGCTAAGGATTTTTTTTTTCGTGCCGCGCAAGCACCATGCAAGCTCTAGAGGCATCAAATAAAACGAAAGGCTCAGTCGAAAGACTGGGCCTTTCGTTTTATCTGTTGTTTGTCGGTGAACGCTCTCCTGAGTAGGACAAATCCGCCGCCCTAGACCTAGGGTACGGGTTTTGCTGCCCGCAAACGGGCTGTTCTGGTGTTGCTAGTTTGTTATCAGAATCGCAGATCCGGCTTCAGCCGGTTTGCCGGCTGAAAGCGCTATTTCTTCCAGAATTGCCATGATTTTTTCCCCACGGGAGGCGTCACTGGCTCCCGTGTTGTCGGCAGCTTTGATTCGATAAGCAGCATCGCCTGTTTCAGGCTGTCTATGTGTGACTGTTGAGCTGTAACAAGTTGTCTCAGGTGTTCAATTTCATGTTCTAGTTGCTTTGTTTTACTGGTTTCACCTGTTCTATTAGGTGTTACATGCTGTTCATCTGTTACATTGTCGATCTGTTCATGGTGAACAGCTTTGAATGCACCAAAAACTCGTAAAAGCTCTGATGTATCTATCTTTTTTACACCGTTTTCATCTGTGCATATGGACAGTTTTCCCTTTGATATGTAACGGTGAACAGTTGTTCTACTTTTGTTTGTTAGTCTTGATGCTTCACTGATAGATACAAGAGCCATAAGAACCTCAGATCCTTCCGTATTTAGCCAGTATGTTCTCTAGTGTGGTTCGTTGTTTTTGCGTGAGCCATGAGAACGAACCATTGAGATCATACTTACTTTGCATGTCACTCAAAAATTTTGCCTCAAAACTGGTGAGCTGAATTTTTGCAGTTAAAGCATCGTGTAGTGTTTTTCTTAGTCCGTTATGTAGGTAGGAATCTGATGTAATGGTTGTTGGTATTTTGTCACCATTCATTTTTATCTGGTTGTTCTCAAGTTCGGTTACGAGATCCATTTGTCTATCTAGTTCAACTTGGAAAATCAACGTATCAGTCGGGCGGCCTCGCTTATCAACCACCAATTTCATATTGCTGTAAGTGTTTAAATCTTTACTTATTGGTTTCAAAACCCATTGGTTAAGCCTTTTAAACTCATGGTAGTTATTTTCAAGCATTAACATGAACTTAAATTCATCAAGGCTAATCTCTATATTTGCCTTGTGAGTTTTCTTTTGTGTTAGTTCTTTTAATAACCACTCATAAATCCTCATAGAGTATTTGTTTTCAAAAGACTTAACATGTTCCAGATTATATTTTATGAATTTTTTTAACTGGAAAAGATAAGGCAATATCTCTTCACTAAAAACTAATTCTAATTTTTCGCTTGAGAACTTGGCATAGTTTGTCCACTGGAAAATCTCAAAGCCTTTAACCAAAGGATTCCTGATTTCCACAGTTCTCGTCATCAGCTCTCTGGTTGCTTTAGCTAATACACCATAAGCATTTTCCCTACTGATGTTCATCATCTGAGCGTATTGGTTATAAGTGAACGATACCGTCCGTTCTTTCCTTGTAGGGTTTTCAATCGTGGGGTTGAGTAGTGCCACACAGCATAAAATTAGCTTGGTTTCATGCTCCGTTAAGTCATAGCGACTAATCGCTAGTTCATTTGCTTTGAAAACAACTAATTCAGACATACATCTCAATTGGTCTAGGTGATTTTAATCACTATACCAATTGAGATGGGCTAGTCAATGATAATTACTAGTCCTTTTCCTTTGAGTTGTGGGTATCTGTAAATTCTGCTAGACCTTTGCTGGAAAACTTGTAAATTCTGCTAGACCCTCTGTAAATTCCGCTAGACCTTTGTGTGTTTTTTTTGTTTATATTCAAGTGGTTATAATTTATAGAATAAAGAAAGAATAAAAAAAGATAAAAAGAATAGATCCCAGCCCTGTGTATAACTCACTACTTTAGTCAGTTCCGCAGTATTACAAAAGGATGTCGCAAACGCTGTTTGCTCCTCTACAAAACAGACCTTAAAACCCTAAAGGCTTAAGTAGCGCCGTCGCAAGCTCCGGCAAATCGCTGAATATTCCTTTTGTCTCCGACCATCAGGCACCTGAGTCGCTGTCTTTTTCGTCACATTCAGTTCGCTGCGCTCACGGCTCTGGCAGTGAATGGGGGTAAATGGCACTACAGGCGCCTTTTATGGATTCATGCAAGGAAACTACCCATAATACAAGAAAAGCCCGTCACGGGCTTCTCAGGGCGTTTTATGGCGGGTCTGCTATGTGGTGCTATCTGACTTTTTGCTGTTCAGCAGTTCCTGCCCTCTGATTTTCCAGTCTGACCACTTCGGATTATCCCGTGACAGGTCATTCAGACTGGCTAATGCACCCAGTAAGGCAGCGGTATCATCAACAGGCTTACCCGTCTTACTGTCCCTAGTGCTTGGATTCTCACCAATAAAAAACGCCCGGCGGCAACCGAGCGTTCTGAACAAATCCAGATGGAGTTCTGAGGTCATTACTGGATCTATCAACAGGAGTCCAAGCGAGCTCTCGAACCCCAGAGTCCCGCTATGTATCCGCTCATGAATTAATTCTTATTACGCCCCGCCCTGCCACTCATCGCAGTACTGTTGTAATTCATTAAGCATTCTGCCGACATGGAAGCCATCACAAACGGCATGATGAACCTGAATCGCCAGCGGCATCAGCACCTTGTCGCCTTGCGTATAATATTTGCCCATGGTGAAAACGGGGGCGAAGAAGTTGTCCATATTGGCCACGTTTAAATCAAAACTGGTGAAACTCACCCAGGGATTGGCTGAGACGAAAAACATATTCTCAATAAACCCTTTAGGGAAATAGGCCAGGTTTTCACCGTAACACGCCACATCTTGCGAATATATGTGTAGAAACTGCCGGAAATCGTCGTGGTATTCACTCCAGAGCGATGAAAACGTTTCAGTTTGCTCATGGAAAACGGTGTAACAAGGGTGAACACTATCCCATATCACCAGCTCACCGTCTTTCATTGCCATACGAAATTCCGGATGAGCATTCATCAGGCGGGCAAGAATGTGAATAAAGGCCGGATAAAACTTGTGCTTATTTTTCTTTACGGTCTTTAAAAAGGCCGTAATATCCAGCTGAACGGTCTGGTTATAGGTACATTGAGCAACTGACTGAAATGCCTCAAAATGTTCTTTACGATGCCATTGGGATATATCAACGGTGGTATATCCAGTGATTTTTTTCTCCATTTTAGCTTCCTTAGCTCCTGAAAATCTCGATAACTCAAAAAATACGCCCGGTAGTGATCTTATTTCATTATGGTGAAAGTTGGAACCTCTTACGTGCCGATCAAGATCAAAGGATCTTCTTGAGATCCCCAGCTGGCAATTCCGAC

Additional supporting information (Tables S10-S13) can be found in the separate Excel file.

**References:**

1 Chan, P. P. & Lowe, T. M. GtRNAdb 2.0: an expanded database of transfer RNA genes identified in complete and draft genomes. *Nucleic Acids Res* **44**, D184-189 (2016). <https://doi.org:10.1093/nar/gkv1309>

2 Dong, H., Nilsson, L. & Kurland, C. G. Co-variation of tRNA abundance and codon usage in *Escherichia coli* at different growth rates. *J Mol Biol* **260**, 649-663 (1996). <https://doi.org:10.1006/jmbi.1996.0428>

3 Murphy, F. V. t. & Ramakrishnan, V. Structure of a purine-purine wobble base pair in the decoding center of the ribosome. *Nat Struct Mol Biol* **11**, 1251-1252 (2004). <https://doi.org:10.1038/nsmb866>

4 Baba, T. *et al.* Construction of *Escherichia coli* K-12 in-frame, single-gene knockout mutants: the Keio collection. *Mol Syst Biol* **2**, 2006 0008 (2006). <https://doi.org:10.1038/msb4100050>

5 Martinez-Garcia, E. *et al.* SEVA 4.0: an update of the Standard European Vector Architecture database for advanced analysis and programming of bacterial phenotypes. *Nucleic Acids Res* **51**, D1558-D1567 (2023). <https://doi.org:10.1093/nar/gkac1059>

6 Napiorkowska, M., Pestalozzi, L., Panke, S., Held, M. & Schmitt, S. High-throughput optimization of recombinant protein production in microfluidic gel beads. *Small* **17**, e2005523 (2021). <https://doi.org:10.1002/smll.202005523>

7 Keseler, I. M. *et al.* EcoCyc: a comprehensive database resource for *Escherichia coli*. *Nucleic Acids Res* **33**, D334-337 (2005). <https://doi.org:10.1093/nar/gki108>
