# Supplementary material for: Systematic analysis of tRNA transcription unit deletions in E. coli reveals insights into tRNA gene essentiality and cellular adaptation
Source: Sci Rep. 2024 Oct 15;14:24102. doi: 10.1038/s41598-024-73407-7 (PMC11480407; doi:10.1038/s41598-024-73407-7)
Supplement: Supplementary file 3 — Supplementary Material 3 [file 41598_2024_73407_MOESM3_ESM.pdf]

Appendix

Systematic Analysis of tRNA Transcription Unit Deletions in *E. coli* Reveals Insights into tRNA Gene Essentiality and Cellular Adaptation

Sanja Tiefenbacher[1], Valérie Pezo[2], Philippe Marlière[3], Tania M. Roberts[1], Sven Panke[1*]

[1] *Bioprocess Laboratory, Department of Biosystems Science and Engineering, ETH Zurich, 4056 Basel, Switzerland*

[2] *Génomique Métabolique, Genoscope, Institut François Jacob, CEA, CNRS, Univ Evry, Université Paris-Saclay, 91057 Evry, France*

[3] *TESSSI, The European Syndicate of Synthetic Scientists and Industrialists, 75002 Paris, France*

*corresponding author: sven.panke@bsse.ethz.ch

The appendix includes uncropped images of agarose gel electrophoresis from the colony PCR analysis.

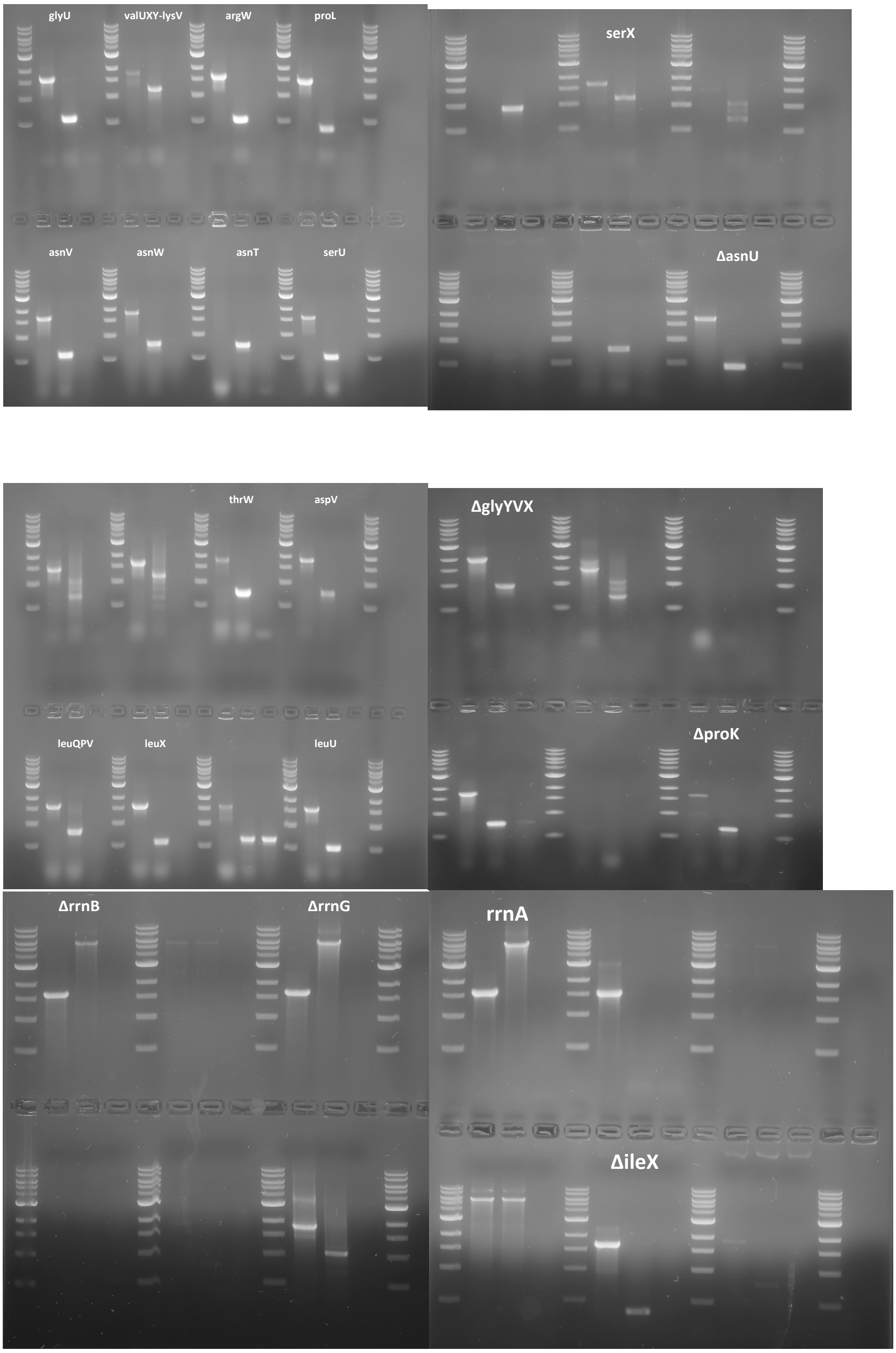

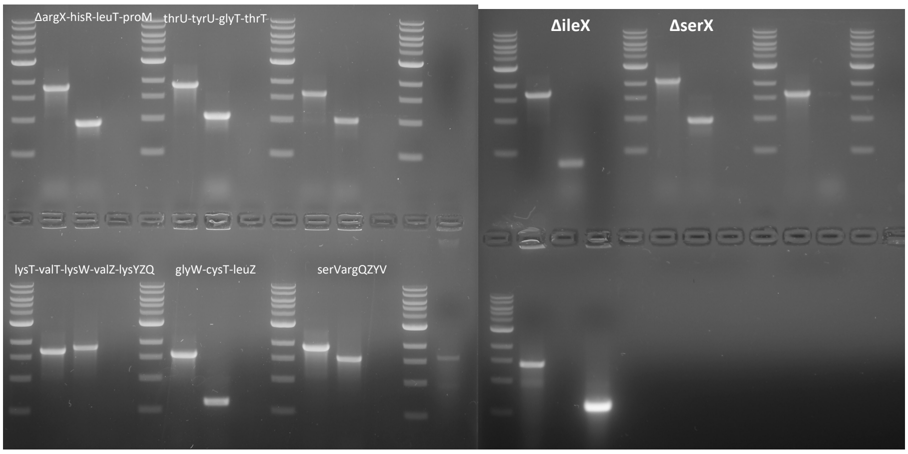
ΔargX-hisR-leuT-proM
thrU-tyrU-glyT-thrT
ΔileX
ΔserX
lysT-valT-lysW-valZ-lysYZQ
glyW-cysT-leuZ
serVargQZYV

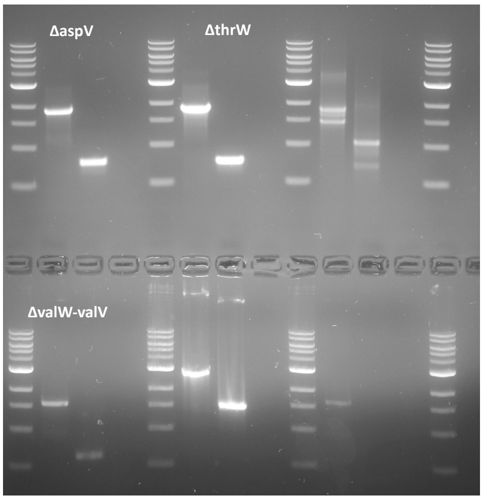
ΔaspV
ΔthrW
ΔvalW-valV

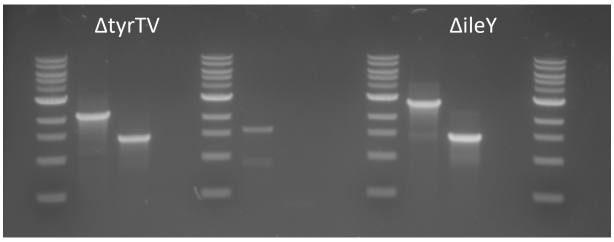
ΔtyrTV
ΔileY

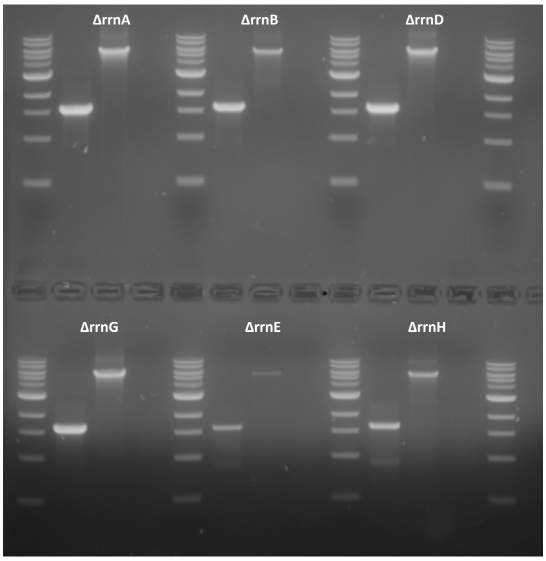
ΔrrnA
ΔrrnB
ΔrrnD
ΔrrnG
ΔrrnE
ΔrrnH

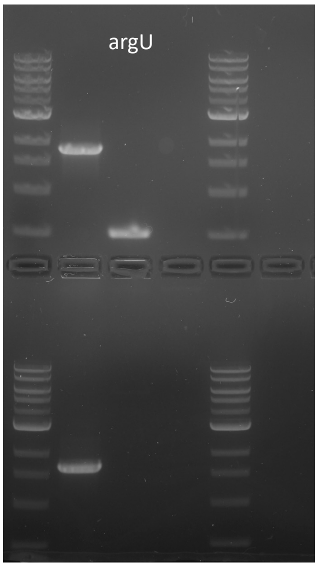
argU

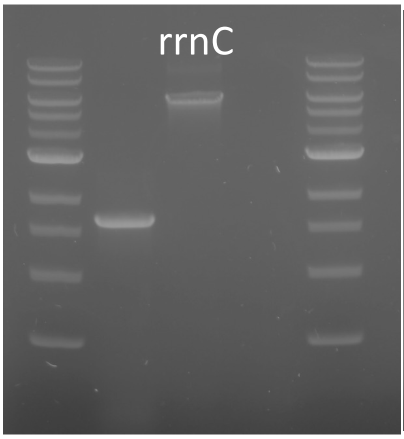
rrnC

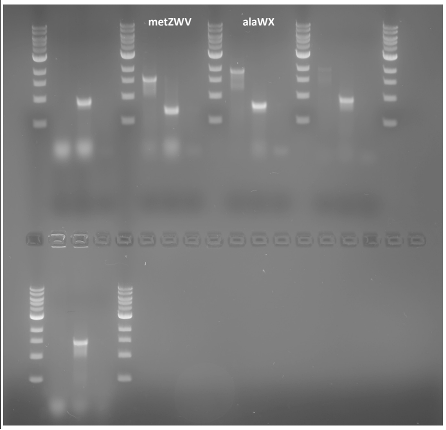
metZWV
alaWX

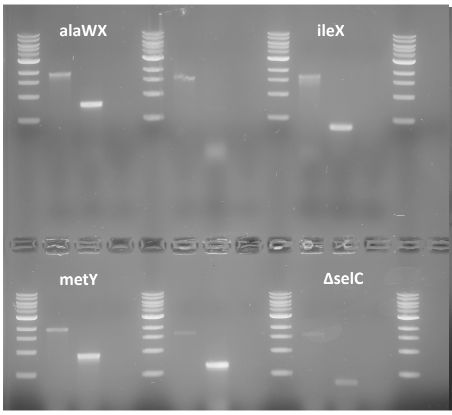
alaWX
ileX
metY
ΔselC

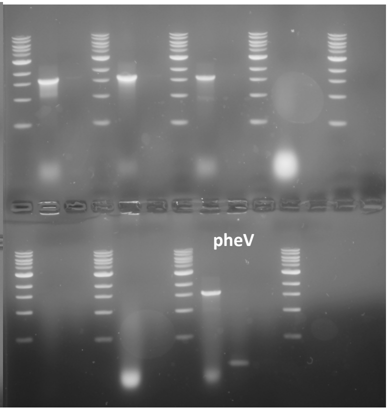
pheV
